# Synergism of IP_3_R and Parkin mutants identifies mitochondrial stress as an early feature of Parkinson's disease

**DOI:** 10.1242/dmm.052146

**Published:** 2026-01-21

**Authors:** Mrudula Dileep, Anamika Sharma, Nandashree Kasturacharya, Syed Kavish Nizami, Steffy Manjila, Ashita Bhan, Gaiti Hasan

**Affiliations:** ^1^ National Centre for Biological Sciences, TIFR, Bengaluru 560065, India; ^2^ Centre for High Impact Neuroscience and Translational Applications (CHINTA), TCG-Crest, Kolkata 700091, India

**Keywords:** Dopaminergic neurons, PPL1, Ca^2+^ homeostasis, Motor function, Flight

## Abstract

Our understanding of mechanisms underlying familial Parkinson's disease (PD) have benefitted from studies in *Drosophila* models of PD. However, in a majority of patients with PD, the disease occurs sporadically, and cellular phenotypes that arise early in sporadic PD are not yet fully understood. A genetic predisposition, arising from variants in pathways that impact dopaminergic neuron health could be one cause of sporadic PD. Here, we studied *Drosophila* with single-copy mutation of the recessive IP_3_R-encoding gene (*itpr*) in combination with a recessive null mutation of the *parkin* gene. Whereas individual mutants appeared normal, in combination, the genes synergised so that flies exhibited flight motor deficits with a focus in a subset of central dopaminergic neurons. Surprisingly, mitophagy and mitochondrial Ca^2+^ were barely affected. Instead, flight motor deficits correlated with elevated levels of mitochondrial H_2_O_2_, and reducing H_2_O_2_ levels by genetic means restored mitochondrial function and flight to a significant extent. This study underlines the importance of mitochondrial oxidative stress as an early phenotype in PD and suggests that humans with recessive variants in either pathway have a higher chance of developing sporadic PD.

## Teaser

**Editor's choice:** Our study reveals that synergism of *itpr* and *parkin* mutants in *Drosophila* leads to flight motor deficits and mitochondrial oxidative stress in dopaminergic neurons, identifying a key feature of early Parkinson's disease.

## INTRODUCTION

Parkinson's disease (PD) is a debilitating neurodegenerative disorder that results in loss of dopaminergic neurons from the substantia nigra pars compacta region of the human brain. The aetiology of PD can be either familial or sporadic (Masato et al., 2019), and is similar in both cases. An understanding of the cellular causes underlying PD pathology comes from a combination of investigations in human patients and model organisms (Hewitt and Whitworth, 2017; Pickrell and Youle, 2015). A well-studied cellular mechanism impacted in a large percentage of familial patients with PD, and modelled in *Drosophila*, is the PINK1/parkin pathway that primarily works to remove defective mitochondria (Pereira et al., 2023). Persistent mitochondrial activity is a hallmark of neuronal cells because they require maintenance of the electrochemical gradient at the plasma membrane and neurotransmitter release at the synapse, both of which are energy-intensive processes that, in turn, lead to the generation of reactive oxygen species (ROS) that can cause mitochondrial damage. When there is loss or reduced activity of the PINK1/parkin pathway, bioenergetically compromised mitochondria accumulate, resulting in neuronal death (Ricke et al., 2020).

Because several mitochondrial dehydrogenases require Ca^2+^ as a co-factor (McCormack and Denton, 1979), changes in neuronal Ca^2+^ signalling, either through activity-dependent mechanisms or through endoplasmic reticulum (ER)-Ca^2+^ release, also target mitochondrial function and have been implicated in PD pathology (Zaichick et al., 2017). Moreover, a recent study identified PINK1/Parkin as indirect regulators of Inositol 1,4,5-trisphosphate receptor (IP_3_R)-mediated ER-Ca^2+^ release (Ham et al., 2023). Based on these studies, we hypothesised that one cause of sporadic PD may be convergent toxicity arising from compromised PINK1/parkin and intracellular Ca^2+^ signalling pathways in dopaminergic neurons. To test this idea, we measured deficits in mitochondrial and cellular function in *Drosophila* when there is partial loss of Parkin and IP_3_R.

Functional interactions between cellular signalling pathways can be identified by measuring genetic interactions of mutants in the individual pathways (Pérez-Pérez et al., 2009). The key is to examine whether the phenotype of the double mutants exceeds the expected phenotype of the single mutants. Previous work has shown that ER-Ca^2+^ release through the IP_3_R is required in the PPL1 cluster of central dopaminergic neurons to maintain the longevity of *Drosophila* flight bouts (Sharma and Hasan, 2020). Flight is also affected in *parkin* mutants, in which apoptotic death of flight muscles was considered as the primary cause (Greene et al., 2003; Park et al., 2006). However, studies have also identified loss of PPL1 dopaminergic neurons in *parkin* mutants (Whitworth et al., 2005; Ham et al., 2023). To understand how the IP_3_R and Parkin proteins might work together to maintain the integrity of flight-promoting dopaminergic neurons, we examined systemic and cellular phenotypes of *Drosophila* mutant combinations for Parkin and IP_3_R where the individual mutants appeared normal.

## RESULTS

### Synergistic loss of function in IP_3_R and Parkin mutant combinations

**Fig. 1.**
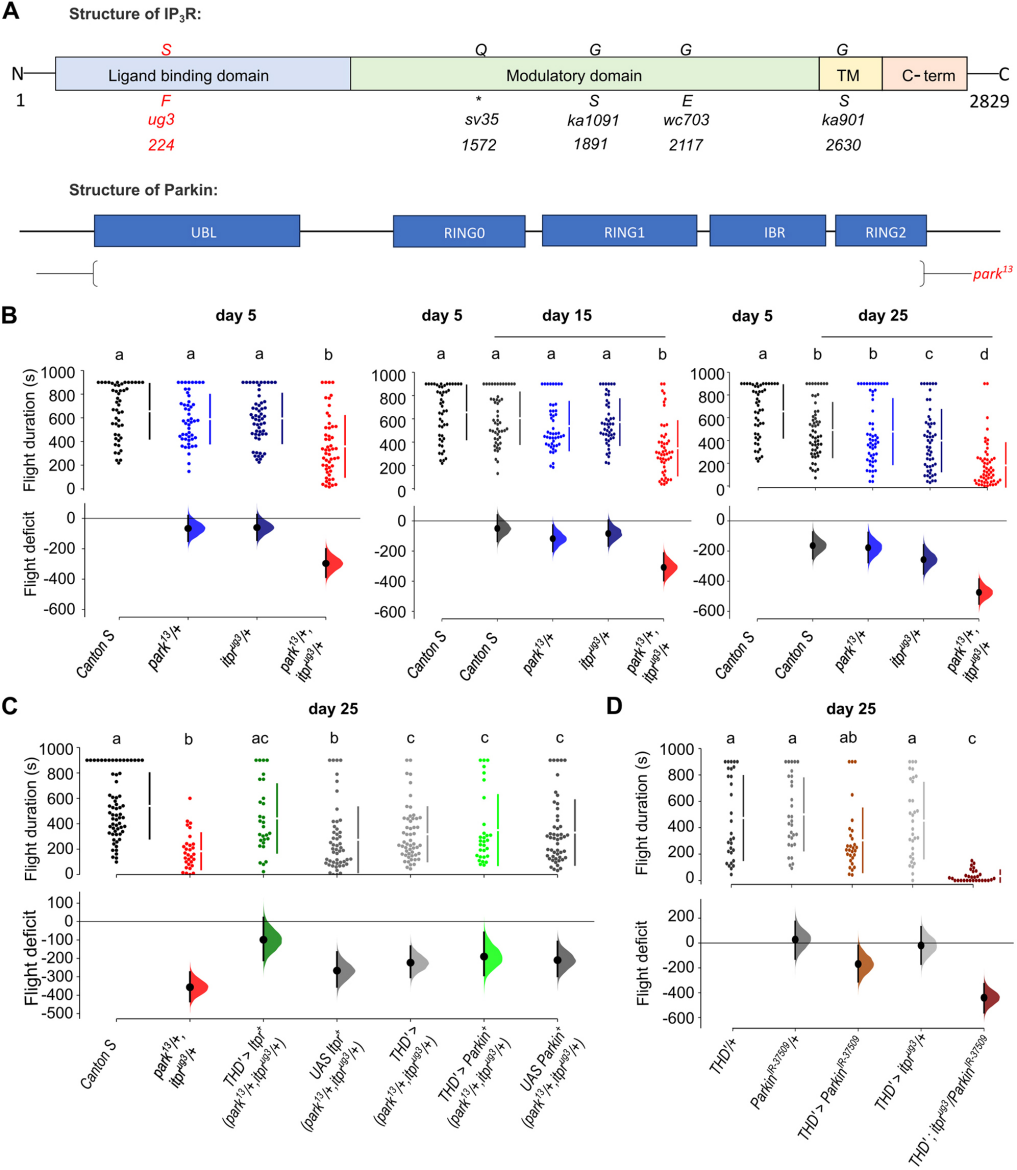
**Age-dependent flight deficits in *park^13^/itpr^ug3^* flies arise from central dopaminergic neurons.** (A) Domain organisation of *Drosophila itpr* and *parkin* genes. The *itpr* gene is depicted with the positions of mutated amino acid residues in the mutant alleles used in this study. *park^13^* is a complete deficiency for *parkin*. The asterisk indicates a stop codon. TM, transmembrane domain. (B) Flight durations of the indicated genotypes at day 5, 15 and 25 are shown as a swarm plot (top) and effect size (bottom). The effect size is depicted as the mean difference in flight (Δ flight) of the indicated genotypes when compared with the control (*Canton S*) at day 5. Each dot in the swarm plot represents the flight time of a single fly. Vertical error bars towards the right of the swarm plots indicate the end of each 95% confidence interval. Comparison of *Canton S* flies (black) at Day 5 (*N*=49), against Day 15 (*N*=50) and Day 25 (*N*=56) *Canton S* flies (dark grey), *park^13^* control (royal blue), *itpr^ug3^* control (navy blue) and the *park^13^/+; itpr^ug3^/+* heteroallelic combination (red). Day 5: *park^13^*/+ (*N*=50), *itpr^ug3^*/+ (*N*=56), *park^13^*/+*, itpr^ug3^*/+ (*N*=53). Day 15: *park^13^/+* (*N*=48), *itpr^ug3^/+* (*N*=47), *park^13^/+, itpr^ug3^/+* (*N*=50). Day 25: *park^13^/+* (*N*=54), *itpr^ug3^/+* (*N*=53), *park^13^/+, itpr^ug3^/+* (*N*=55). Test for significance was interpreted using a non-parametric, Mann–Whitney test. (C) Expression of an *Itpr^+^* transgene in the PPL1+PPM3 clusters of central dopaminergic neurons, marked by *THD′GAL4*, rescues flight deficits (dark green) of *park^13^/+; itpr^ug3^/+* (red) at day 25. Presence of individual transgenes, *THD′GAL4* or *Itpr^+^ in park^13^/itpr^ug3^* improve the flight times (grey shades) but remain significantly different from those of the *Canton S* control at day 25. Rescue of flight was not significant upon expression of *Parkin^+^* in *THD′*-marked dopaminergic neurons of *park^13^/itpr^ug3^ (THD′>Parkin^+^; park^13^/+, itpr^ug3^/+*; light green) and was similar to flight rescue seen upon expression of individual transgenes, either *THD′GAL4* or *Parkin^+^* (grey shades). *THD′>Itpr^+^; park^13^/+, itpr^ug3^/+* (*N*=29) in dark green. *THD′>Parkin^+^; park^13^/+, itpr^ug3^/+* (*N*=30) in light green*, UAS Itpr^+^; park^13^/+, itpr^ug3^/+* (*N*=42), *THD′; park^13^/+, itpr^ug3^/+* (*N*=43), *UAS Parkin^+^; park^13^/+, itpr^ug3^/+* (*N*=37). Test for significance was interpreted using a non-parametric, Mann–Whitney test. (D) A weak flight deficit obtained by expression of a *ParkinRNAi* (*parkin^IR-37509^*) in THD′-marked neurons (light brown) is enhanced significantly in the background of one copy of *itpr^ug3^* (*THD′>parkin^IR-37509^/itpr^ug3^*; dark brown). *N*=29 for every genotype. Test for significance was interpreted using a non-parametric, Mann–Whitney test. Individual comparisons of genotypes with their *P*-values are provided in Table S1. Lower-case letters above each genotype, when different, represent significant differences from flight times of other genotypes (*P*<0.05).

To examine whether the *itpr* (encoding IP_3_R) and *parkin* genes interact functionally, we tested heteroallelic combinations of a *parkin* null mutant *park^13^* (Greene et al., 2003) with various *itpr* mutants (Fig. 1A; Joshi et al., 2004). The *itpr* mutants tested included the truncated null allele *itpr^sv35^* and *itpr^ka901^*, a mutation in the Ca^2+^ channel that creates a ‘pore dead’ protein (Srikanth et al., 2006; Fig. S1A). The final progeny tested bear one mutated allele for each gene. We found that one copy of *park^13^* with one copy of stronger *itpr* alleles, *itpr^sv35^* and *itpr^ka901^*, were lethal at second-instar larval stage (Fig. S1A and Table S1), indicating a strong genetic interaction between *itpr* and *parkin* genes. Lethality was also observed with a second parkin allele *park^25^* in combination with *itpr^ka901^* (*park^25^/+, itpr^ka901^/+*; Table S1). All heterozygote controls, including *park^13^*/+, *park^25^/+*, *itpr^sv35^*/+ and *itpr^ka901^*/+, were viable.

To further investigate the genetic interaction observed between IP_3_R- and Parkin-encoding genes, we began by identifying adult viable combinations of *parkin* and *itpr* mutant alleles. Single copies of three weaker *itpr* alleles tested – *itpr^wc703^*, *itpr^ka1091^* and *itpr^ug3^* (Joshi et al., 2004) – were viable in combination with *park^13^*/+ (Fig. S1B). Previous studies have demonstrated that recessive alleles of either *Drosophila itpr* or *parkin* exhibit flight deficits (Banerjee et al., 2004; Park et al., 2006). Therefore, we tested flight in flies carrying one copy each of the *Parkin* null allele, *park^13^* and a well-characterised *itpr* allele, *itpr^ug3^* (Srikanth et al., 2006), which is viable in several heteroallelic combinations (Joshi et al., 2004). *park^13^/itpr^ug3^* flies exhibited significant flight deficits starting at day 5, which were further exaggerated by day 25, compared with either wild-type [WT; *Canton S* (*CS*)] flies or *park^13^/+* and *itpr^ug3^/+* heterozygote controls (Fig. 1B). Thus, the synergism between Parkin and IP_3_R affects adult motor function in an age-dependent manner. In an independent experiment, we demonstrated a flight deficit, indicating a genetic interaction, in heterozygotes of a mutant allele of the Parkin-interacting gene, *pink^1B9^* (Park et al., 2006), and *itpr^ug3^* (Fig. S1F).

### Parkin and IP_3_R function in flight is required in a subset of adult dopaminergic neurons

Previous studies have identified neuronal and muscle deficits in *parkin* mutants (Park et al., 2006). In agreement with these published data, the observed flight deficit in *park^13^/itpr^ug3^* animals could be partially rescued by expression of an *Itpr^+^* transgene in either neurons or muscles using appropriate tissue-specific *GAL4* strains, *nsybGAL4* and *DMefGAL4* (Fig. S1C). Locomotor defects observed in *parkin* mutants are associated with central dopaminergic neurons (Sang et al., 2007). Therefore, we tested whether the flight deficit in *park^13^/itpr^ug3^* animals arises from flight-promoting central dopaminergic neurons (fpDANs), marked by *THD′GAL4* (Fig. S2), and identified earlier as requiring IP_3_R function (Sharma and Hasan, 2020) and intracellular Ca^2+^ signalling (Mitra et al., 2024). The fpDANs include the PPL1 and PPM3 clusters of adult dopaminergic neurons (Fig. S2).

Overexpression of IP_3_R in fpDANs of *park^13^/itpr^ug3^* animals rescued flight deficits to a significant extent compared to mutant and control genotypes (Fig. 1C; Table S1). Rescue experiments, performed in parallel, by overexpression of *Parkin^+^* gave ambiguous results. Although muscle expression of *Parkin^+^* rescued the flight deficit of *park^13^/itpr^ug3^* to a significant extent (Fig. S1C and Table S1), flight deficits upon *Parkin^+^* expression by pan-neuronal *GAL4* and dopaminergic neuronal *GAL4* strains were similar among mutant control and rescue strains (Fig. 1C; Fig. S1C). Requirement for Parkin in adult neurons was tested further by knockdown using a *GAL4* strain that expresses in a majority of dopaminergic neurons (*THGAL4*; Friggi-Grelin et al., 2003) including the fpDANs, and three different *parkin* RNA interference (RNAi) strains. In all cases, significant flight deficits were observed (Fig. S1D and Table S1), indicating that Parkin is required in dopaminergic neurons for flight. Next, we tested whether the minor flight deficit observed by knockdown of *parkin* in fpDANs with *parkin^IR-37509^* could be enhanced in the genetic background of one copy of the IP_3_R-encoding mutant, *itpr^ug3^*. Indeed, the presence of *itpr^ug3^* led to a significant decrease in the duration of flight bouts when compared to expression of *parkin^IR-37509^* on its own (Fig. 1D and Table S1). The rescue and knockdown data together support a requirement for *parkin* and *itpr* function in fpDANs, in which overexpression of *Itpr^+^* can rescue the synergistic deficit of a single copy of *park^13^* in the presence of a single copy of *itpr^ug3^*. However, flight deficits observed in a *parkin* mutant combination (*park^13^/park^1^*) were not rescued by expression of *Itpr*^+^ in fpDANs (Fig. S1E). IP_3_R is thus insufficient to rescue cellular deficits associated with loss of Parkin alone, suggesting that the cellular deficits associated with *park^13^/itpr^ug3^* are either distinct or, more likely, a smaller subset of the deficits arising from loss of Parkin.

**Fig. 2.**
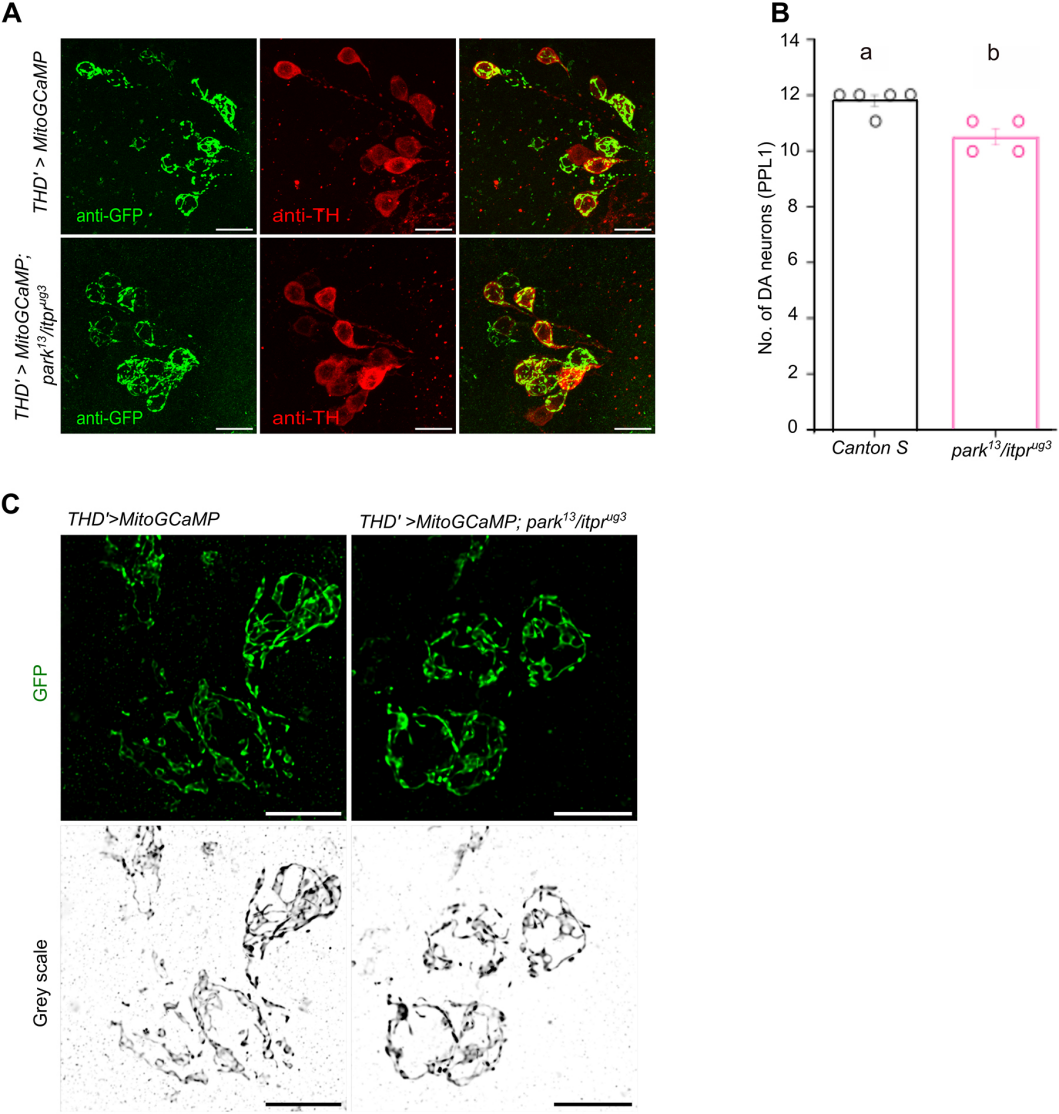
**Quantification and morphology of dopaminergic (DA) neurons in *park^13^/itpr^ug3^* animals.** (A) Loss of DA neurons in the PPL1 cluster of *park^13^/itpr^ug3^* animals. DA neurons of the PPL1 cluster visualised by expression of MitoGCaMP3 in adult *Drosophila* brains of controls (*THD′>MitoGCaMP*3) and *park^13^/itpr^ug3^* (*THD′>MitoGCaMP*3; *park^13^/itpr^ug3^*) animals immunostained with anti-GFP (green) for THD′ neurons) and anti-Tyrosine hydroxylase (anti-TH; red) to visualise all DA neurons. Scale bars: 10 μm. (B) Numbers of DA neurons (PPL1 cluster) appear to be reduced in *park^13^/itpr^ug3^* animals as visualised in brain hemi-segments in A. PPL1 cells were identified by immunostaining with anti-GFP and anti-TH. *Canton S* (*N*=5 brains) is represented in black, and *THD′>MitoGCaMP*3; *park^13^/itpr^ug3^* (*N*=4 brains) is represented in pink. A single hemi-brain was counted from each brain. Mean±s.e.m. for *Canton S* is 11.8±0.2 and for *park^13^/itpr^ug3^* is 10.5±0.3. Significance (*P*<0.05) was tested by a two-tailed *t*-test assuming two-sample unequal variances. Different lower-case letters indicate significant difference in *P*-values. Individual comparisons of genotypes with their *P*-values are provided in Table S4. (C) Mitochondrial numbers and morphology appear normal in DA neurons of the PPL1 cluster of *park^13^/itpr^ug3^* animals. Mitochondria were visualised in the indicated genotypes after immunostaining with anti-GFP. The GFP images were converted to grey scale to better visualise mitochondrial morphology. Scale bars: 5 μm.

Loss of either Parkin or IP_3_R function can lead to partial loss of the PPL1 dopaminergic neurons in fly brains (Ham et al., 2023). Therefore, we investigated whether the age-dependent flight deficits observed in *park^13^/itpr^ug3^* flies correlate with loss of PPL1 cells, earlier identified as requiring IP_3_R for extended flight bouts (Sharma and Hasan, 2020; Fig. S2A). The number of PPL1 dopaminergic neurons appeared significantly reduced, from 11 to 12 pairs in the hemi-brain of control animals at day 25 to ten to 11 pairs in *park^13^/itpr^ug3^* animals (Fig. 2A,B). We were unable to establish the exact identity of the missing pairs of cells. Their loss may be a contributory factor in the flight deficits observed in Fig. 1. Mitochondrial morphology in dopaminergic neurons of the PPL1 cluster appeared similar in WT (*CS*) and *park^13^/itpr^ug3^* brains at day 25 (Fig. 2C). Next, we measured the number of ER-mitochondrial contacts in neurites of PPL1 neurons from control and *park^13^/itpr^ug3^* brains (Fig. S2B). A small, but significant change, in ER-mitochondrial contacts was observed in THD′ neurites between controls and *itpr^ug3^/+* animals. This was further enhanced in *park^13^/itpr^ug3^* animals (Fig. S2B).

### Mitochondrial Ca^2+^ uptake and mitophagy appear normal in *park^13^/itpr^ug3^* animals

**Fig. 3.**
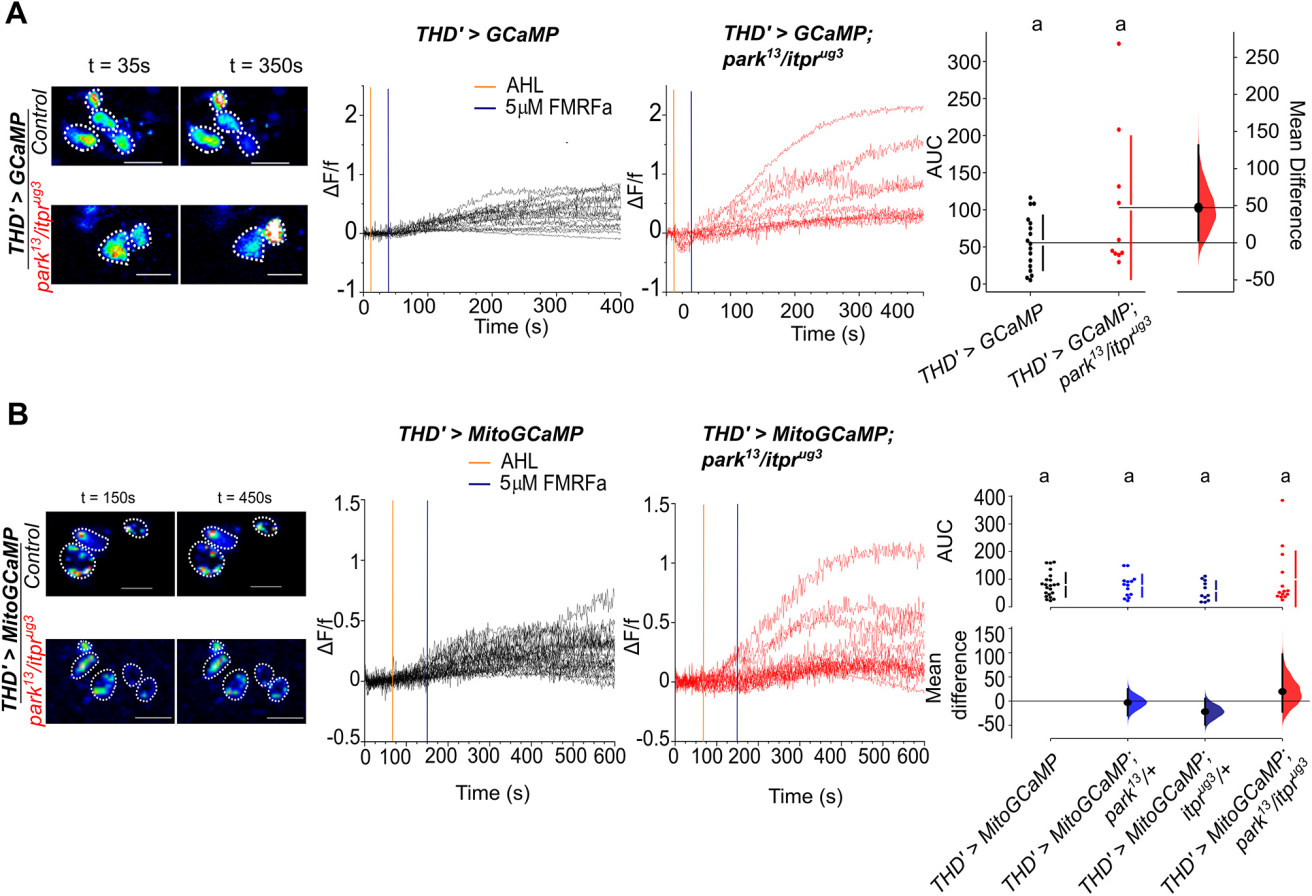
**Endoplasmic reticulum (ER) Ca^2+^ release and mitochondrial Ca^2+^ uptake appear normal in *park^13^/itpr^ug3^* animals.** (A) ER-Ca^2+^ release judged by *UASGCaMP6m* between wild-type (WT) flies and the heteroallelic mutant combination in adult DA neurons. Representative confocal images (left) of the neurons before 5 μM FMRFa addition at 35 s and after 350 s of the indicated genotypes and their individual traces (middle) of WT flies (black) and the heteroallelic mutant combination (red) in live imaging at the indicated time intervals after stimulation with 5 mM FMRFa. Traces represent normalised changes in GCaMP6 m fluorescence (ΔF/f) in the subset of DA neurons. Scale bars: 10 μm. Dotted lines outline individual cells. The images were acquired at 1 frame per second (FPS). The area under the curve (AUC) taken for each individual trace of the mentioned genotypes for individual cells of DA neurons denoting ER-Ca^2+^ release (right). The relative mean difference between the WT in black (*N*=5 brains, *n*=11 cells) and the heteroallelic mutant in red (*N*=4 brains, *n*=10) is non-significant as judged by the non-parametric Mann–Whitney test. (B) Mitochondrial Ca^2+^ uptake as judged by *UAS MitoGCaMP3* between WT flies and the heteroallelic mutant in adult DA neurons. Representative confocal images (left) of the neurons before 5 μM FMRFa addition at 150 s seconds and after 450 s of the indicated genotypes and their individual traces (middle) of WT flies (black) and the heteroallelic mutant combination (red) in live imaging at the indicated time intervals after stimulation with 5 mM FMRFa. Traces represent normalised changes in MitoGCaMP3 fluorescence (ΔF/f) in the subset of DA neurons. Scale bars: 10 μm. The images were acquired at 1.5 FPS. The AUC taken for each individual trace of the mentioned genotypes for individual cells of DA neurons denoting mitochondrial Ca^+2^ uptake (right). The relative mean difference between the WT (black; *N*=6 brains, *n*=24 cells), *park^13^* control (light blue; *N*=7 brains, *n*=19 cells), *itpr^ug3^* control (navy blue; *N*=6, brains, *n*=11 cells) and heteroallelic mutant (red; *N*=6 brains, *n*=14 cells) is non-significant as judged by the non-parametric Mann–Whitney test. Individual comparisons of genotypes with their *P*-values are provided in Table S6. Different lower-case letters represent significant differences in Ca^2+^ responses compared to other genotypes.

Among its many cellular functions, Parkin is also a negative regulator of Ca^2+^ release through IP_3_R (Ham et al., 2023) and Ca^2+^ released by IP_3_R enters the mitochondria at ER-mitochondrial contact sites (Katona et al., 2022; Rossi et al., 2019). Therefore, we tested cytosolic and mitochondrial Ca^2+^ responses of PPL1 fpDANs in flies aged 25 days (Fig. 3; Fig. S3A,B). *Ex vivo* brains of the appropriate genotypes were stimulated with an agonist, the neuropeptide FMRFamide (FMRFa), known to induce ER-Ca^2+^ release through IP_3_R in PPL1 neurons (Ravi et al., 2018). Both cytosolic and mitochondrial Ca^2+^ responses of *park^13^/itpr^ug3^* neurons exhibited minor aberrations, with some cells showing exaggerated Ca^2+^ release and mitochondrial Ca^2+^ entry. However, the average response did not appear significantly different from either WT (*CS*) or heterozygotic (*park^13^/+* and *itpr^ug3^/+*) controls (Fig. 3; Fig. S3A). The addition of FMRFa also elicited a reduction in mitochondrial and cytosolic Ca^2+^, which appeared similar in controls and heterozygous single and double mutant conditions (Fig. S3C,E,F). The cellular mechanism by which FMRFa reduces cytosolic and mitochondrial Ca^2+^ in a few cells was not investigated further, but is presumably due to coupling of FMRFa, in certain cells of the PPL1 cluster, to an inhibitory signalling mechanism.

**Fig. 4.**
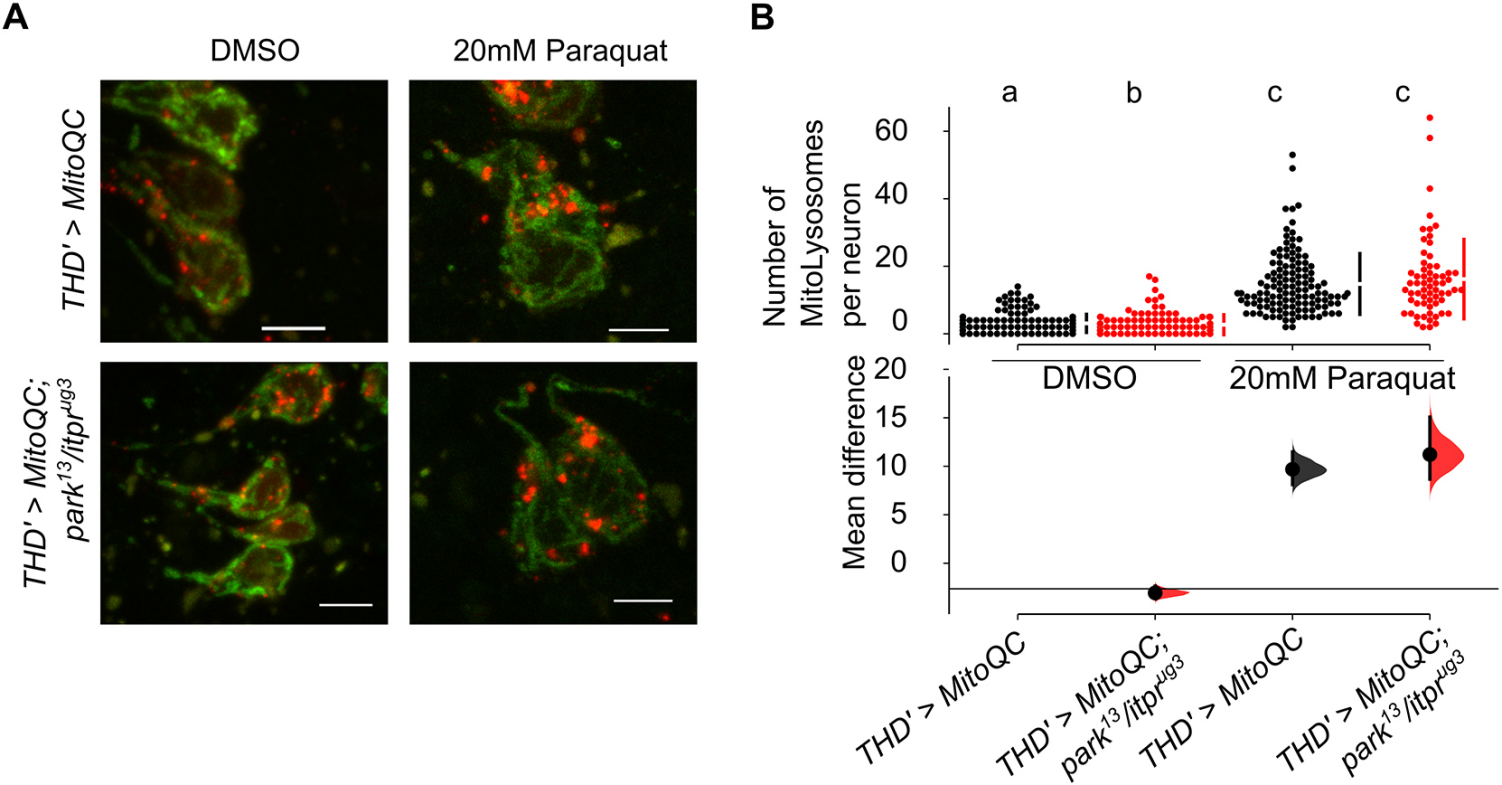
**Mitophagy appears normal in *park^13^/itpr^ug3^* animals.** (A) PPL1 cells were visualised by expression of the mitophagy reporter MitoQC in the indicated genotypes. The images are confocal stacks of dissected 25-day-old adult fly brains treated with DMSO (left column) and paraquat (right column). Scale bars: 5 μm. (B) Mitolysosomes (red) were quantified by performing manual counts from a minimum of five brains of each condition and genotype. Dots represent the number of mitolysosomes (red) in individual cells (*n*>60). Basal mitophagy appears significantly lower in *park^13^/itpr^ug3^* animals than in control (*THD'>MitoQC*) (*P*<0.05, Mann–Whitney test). Paraquat treatment (20 mM) results in higher mitophagy in control (*THD′>MitoQC)* and experimental conditions (*THD′>MitoQC park^13^/itpr^ug3^*). *THD′>MitoQC* (*N*=12, *n*=182), *THD′>MitoQC park^13^/itpr^ug3^* (*N*=10, *n*=136); with 20 mM paraquat, *THD′>MitoQC* (*N*=8, *n*=125), *THD′>MitoQC park^13^/itpr^ug3^* (*N*=5, *n*=64). Tests for significance were interpreted using a non-parametric, Mann–Whitney test. Individual comparisons of genotypes with their *P*-values are provided in Table S7. Different lower-case letters represent significant differences from mitophagy in other genotypes.

A key function of Parkin is to ubiquitinate defective mitochondria, resulting in their removal through mitophagy (Ge et al., 2020; Shimura et al., 2000; Walden and Martinez-Torres, 2012). Loss of Parkin function in dopaminergic neurons thus leads to impaired mitophagy (Cornelissen et al., 2018). In PPL1 neurons from *park^13^/itpr^ug3^* brains, mitophagy was measured by a fluorescent reporter, MitoQC (Montava-Garriga et al., 2020). Basal mitophagy levels appeared somewhat reduced in *park^13^/itpr^ug3^* brains (Fig. 4B). Upon feeding paraquat, a chemical that induced oxidative stress, as expected, there was an increase in mitophagy. However, the elevation appeared similar in control and *park^13^/itpr^ug3^* brains (Fig. 4; Table S4), indicating that the key to neuronal dysfunction in *park^13^/itpr^ug3^* brains is unlikely to be reduced mitophagy.

### *park^13^* and *itpr^ug3^* synergise to elevate mitochondrial H_2_O_2_

**Fig. 5.**
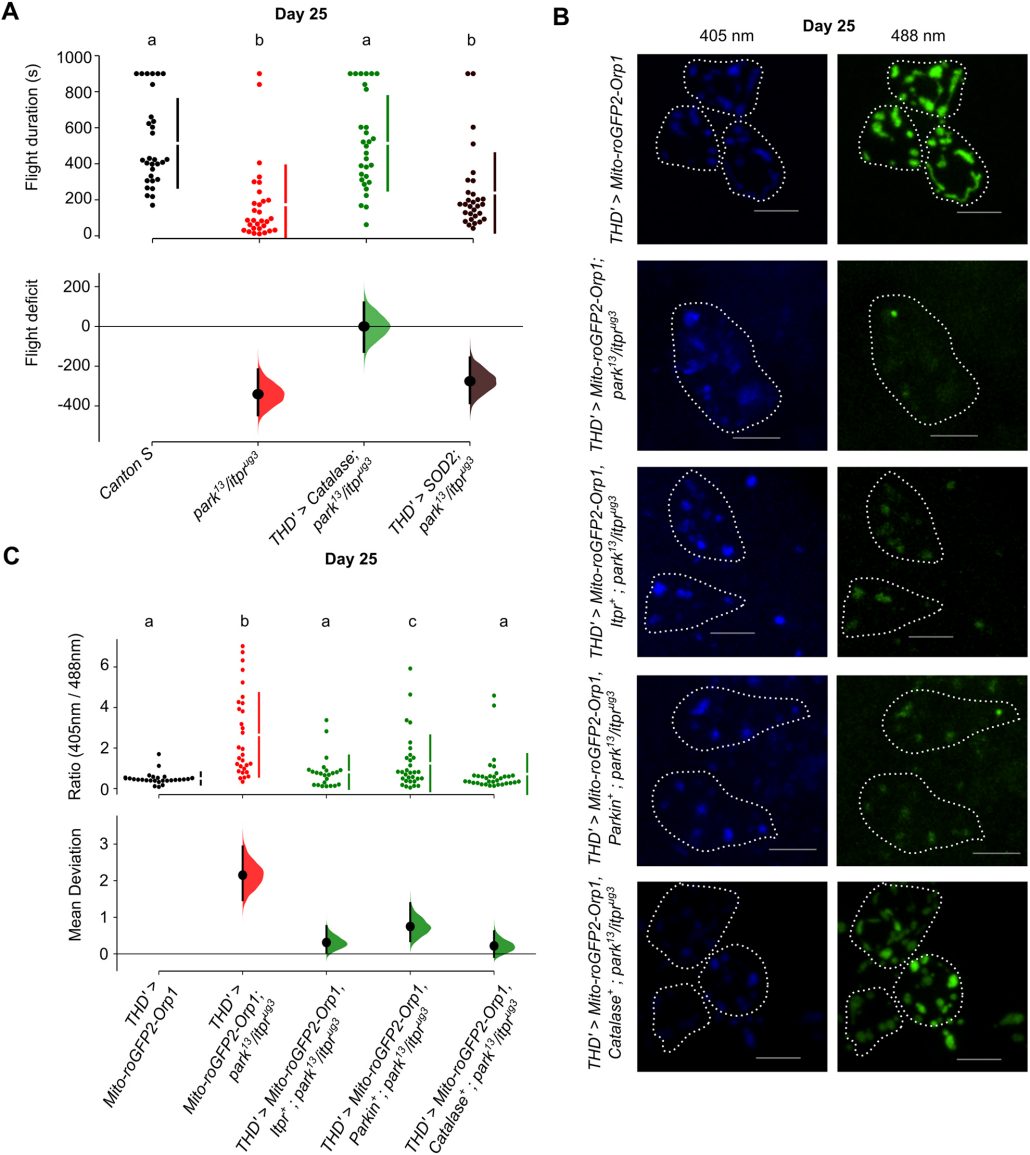
**Flight deficits in *park^13^/itpr^ug3^* animals arise from excess H_2_O_2_ in PPL1 DA neurons.** (A) Overexpression of *Catalase^+^* (green) in *THD′*-marked DA neurons rescues flight deficits in *park^13^/itpr^ug3^* animals. Overexpression of mitochondrial *Sod2^+^* (brown) did not rescue flight deficits. The swarm plots depict flight duration of individual flies of the indicated genotypes at 25 days, where different letters indicate significant change as calculated by the Mann–Whitney test (*P*<0.05). Panel below shows the effect size as compared to the WT control, *Canton S*. *N*=30 for each genotype tested. (B) PPL1 neurons in *park^13^/itpr^ug3^* flies exhibit higher mitochondrial H_2_O_2_ that is rescued by expression of *Catalase^+^* and *Itpr^+^.* Representative images of PPL1 DA neurons obtained from confocal sections of brains of control and *park^13^/itpr^ug3^* adults. Mito-roGFP2-Orp1 is a fluorescent sensor for determining H_2_O_2_ levels. Images at 405 nM measure oxidised GFP in the mitochondria, whereas images at 488 nm measure GFP. Dotted lines outline individual cells. Scale bars: 20 μm. (C) Quantification of H_2_O_2_ levels in the PPL1 DA neuron cluster of individual brains (*N*). Swarm plots demonstrating the ratio of fluorescence at 405 nm to 488 nm, in the mentioned genotypes, are shown on top. Each dot represents the ratio from a single cell (*n*). Different letters represent significant difference (*P*<0.05, Mann–Whitney test). The lower panel shows the effect size in the indicated genotypes compared to that in the control genotype, *THD′> Mito-roGFP2-Orp1*. *THD′>Mito-roGFP2-Orp1* (*N*=6, *n*=28), *THD′G>Mito-roGFP2-Orp1*; *park^13^/itpr^ug3^* (*N*=7, *n*=30), *THD′>Mito-roGFP2-Orp1*; *Itpr^+^, park^13^/itpr^ug3^* (*N*=7, *n*=24), *THD′>Mito-roGFP2-Orp1*; *Parkin^+^, park^13^/itpr^ug3^* (*N*=5, *n*=31), *THD′>Mito-roGFP2-Orp1*; *Catalase^+^, park^13^/itpr^ug3^* (*N*=7, *n*=33). Individual comparisons of genotypes with their *P*-values are provided in Table S8. Different letters represent significant differences from flight times and H_2_O_2_ levels of other genotypes.

Among the cellular properties of dopaminergic neurons that are impacted negatively by mutations that cause PD, including mutations in Parkin, is the ability to handle mitochondrial stress (Burbulla et al., 2017; Praschberger et al., 2023; Xiao et al., 2017; Yin et al., 2018). Moreover, Ca^2+^ transfer at ER-mitochondrial membrane contact sites requires IP_3_R, and small changes in mitochondrial Ca^2+^ also leads to oxidative stress (Baev et al., 2022; Ricke et al., 2020). Therefore, we hypothesised that synergistic dysfunction observed in *park^13^/itpr^ug3^* neurons arises from mitochondrial oxidative stress, which would manifest as excess ROS (O_2_^−^) that undergo conversion to H_2_O_2_. This idea was tested by overexpression of transgenes that encode enzymes for alleviating mitochondrial oxidative stress in fpDANs. Overexpression of *Catalase^+^*, required for conversion of H_2_O_2_ to H_2_O and O_2_ (Rakotoarisoa et al., 2019), in the fpDAN subset of *park^13^/itpr^ug3^* flies rescued flight in 25-day-old flies to a significant extent (Fig. 5A; Fig. S4A). However, expression of *Sod2*, the mitochondrial superoxide dismutase that converts superoxide radicals (O_2_^−^) to H_2_O_2_ did not rescue flight deficits (Fig. 5A). Flight deficits were also observed when a single copy of *park^25^* (Greene et al., 2003) was placed in combination with either an *itpr^DN^* transgene (Sharma and Hasan, 2020; Fig. S4B) or *itpr^ug3^* (Fig. S4C). Significant rescue of flight deficits was observed in flies of the genotype *TH; park^25^/itpr^DN^* and *THD′; park^25^/itpr^ug3^* upon overexpression of *Catalase^+^* in dopaminergic neurons (Fig. S4B,C and Table S7). Minor deficits in Parkin and IP_3_R function thus appear to synergise and raise the level of H_2_O_2_ in fpDANs. This idea was tested next.

H_2_O_2_ levels were measured by expression of a fluorescent sensor, *Mito-roGFP2-Orp1* (Albrecht et al., 2011) in the mitochondria of fpDANs. Here, *Orp1* mediates the oxidation of a redox-sensitive GFP (*roGFP2*) in the presence of H_2_O_2_. Relative levels of H_2_O_2_ were assessed by determining the ratio of emitted fluorescence (405/488 nm) of non-oxidised and oxidised GFP, respectively, in the PPL1 subset of fpDANs. Levels of H_2_O_2_ were significantly elevated in the PPL1 neurons of *park^13^/itpr^ug3^* flies aged 5 days (Table S5 and Fig.  S4D) compared with PPL1 neurons of WT flies. A further elevation of H_2_O_2_ was observed in older (25 days) *park^13^/itpr^ug3^* flies (Fig. 5B; Fig. S4E). Overexpression of *Catalase^+^* and *Itpr^+^* in PPL1 neurons of *park^13^/itpr^ug3^* animals reversed the excess H_2_O_2_ to control levels, whereas overexpression of *Parkin^+^* resulted in a partial reversal. This aligns with our findings in Fig. 1C in which overexpression of *Itpr^+^* resulted in better rescue of flight deficits of 25 day compared to overexpression of *Parkin^+^* (Fig. 5B,C; Table S5). These data support the idea that accumulation of toxic levels of H_2_O_2_ in the PPL1 fpDANs is a causative feature for the observed flight deficits in *park^13^/itpr^ug3^* flies.

### *park^13^/itpr^ug3^* brains exhibit high oxidation of dopamine to DOPAC

**Fig. 6.**
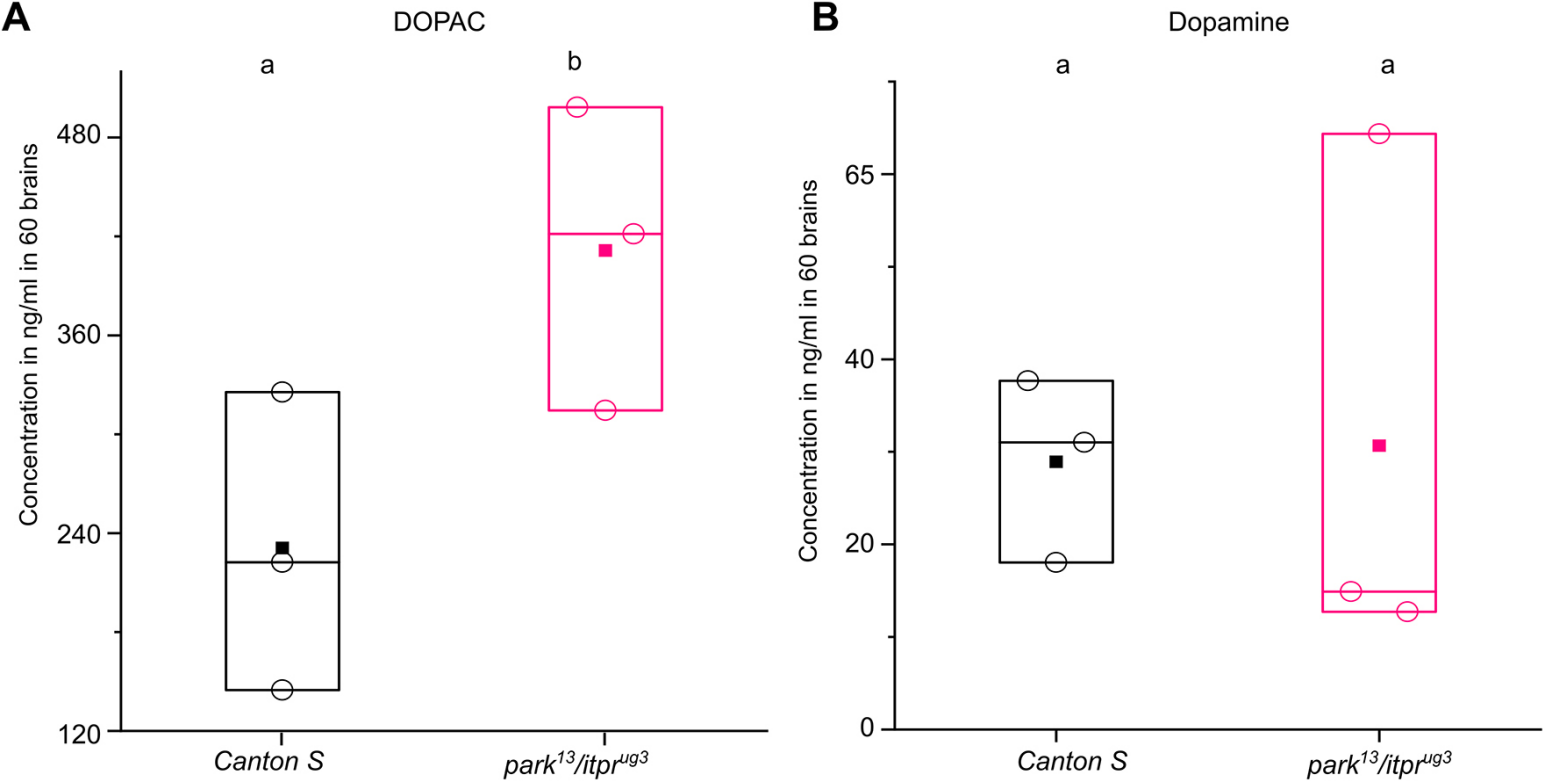
**Increased 3,4-dihydroxyphenylacetic acid (DOPAC) levels in *park^13^/itpr^ug3^* animals.** (A) DOPAC levels in the dissected brains of *park^13^/itpr^ug3^* animals are significantly higher than those in *Canton S* animals. Quantification of DOPAC concentration using mass spectrometry. Box plots representing concentration of DOPAC in three replicates. Each dot represents analysis from a single set containing *N*=60 adult fly brains of *Canton S* (black) and *park^13^/itpr^ug3^* (pink) genotypes. The (mean±s.e.m.) values include *Canton S* (230.8976±74.0223 ng/ml), and *park^13^/itpr^ug3^* (411.4315±75.4461 ng/ml). (B) Dopamine levels in the *park^13^/itpr^ug3^* animal brains appear to be similar to those in *Canton S* animal brains. Quantification of dopamine concentration using mass spectrometry. Box plots representing concentration of dopamine in three replicates. Each dot represents analysis from a single set containing *N*=60 adult fly brains of *Canton S* (black) and *park^13^/itpr^ug3^* (pink) genotypes. The mean (±s.e.m.) values include *Canton S* (28.9±8.15 ng/ml) and *park^13^/itpr^ug3^* (30.6±23.9 ng/ml). Different lower-case letters indicate the level of significance as tested by unpaired *t*-test for two-sample means. *P*<0.05. Individual comparisons of genotypes with their *P*-values are provided in Table S10.

The presence of excess H_2_O_2_ in *Drosophila* dopaminergic neurons led us to investigate its source(s). Previous work has identified electrons leaking from the electron transport chain and related metabolic enzymes as a source of O_2_^−^ and H_2_O_2_ in *Drosophila* (Miwa et al., 2003). Mammalian dopamine neurons can oxidise dopamine at the outer mitochondrial membrane to generate H_2_O_2_ that functions as an electron donor to complex IV of the electron transport chain (Graves et al., 2020). This reaction is catalysed by monoamine oxidase (MAO), which drives oxidation of dopamine to 3,4-dihydroxyphenylacetaldehyde (DOPAL), which is subsequently converted to 3,4-dihydroxyphenylacetic acid (DOPAC). Electrons generated by this mechanism further support the high bioenergetic demand of dopamine release. Thus, excess H_2_O_2_ helps generate ATP, required for the activity of dopaminergic neurons (Chen and Jonas, 2020). To investigate dopamine oxidation by MAO as a possible source of excess H_2_O_2_ in *park^13^/itpr^ug3^*, we quantified levels of dopamine and DOPAC in dissected fly brains using mass spectrometry. A significant increase in the levels of DOPAC was observed in 25-day-old *park^13^/itpr^ug3^* flies compared to those in age-matched control *CS* flies (Fig. 6; Fig. S5A-D, Tables S8, S9). Levels of dopamine appeared variable in the control flies and were not significantly altered in *park^13^/itpr^ug3^* flies.

### ATP production upon depolarisation is compromised in dopaminergic neurons of *park^13^/itpr^ug3^* animals

**Fig. 7.**
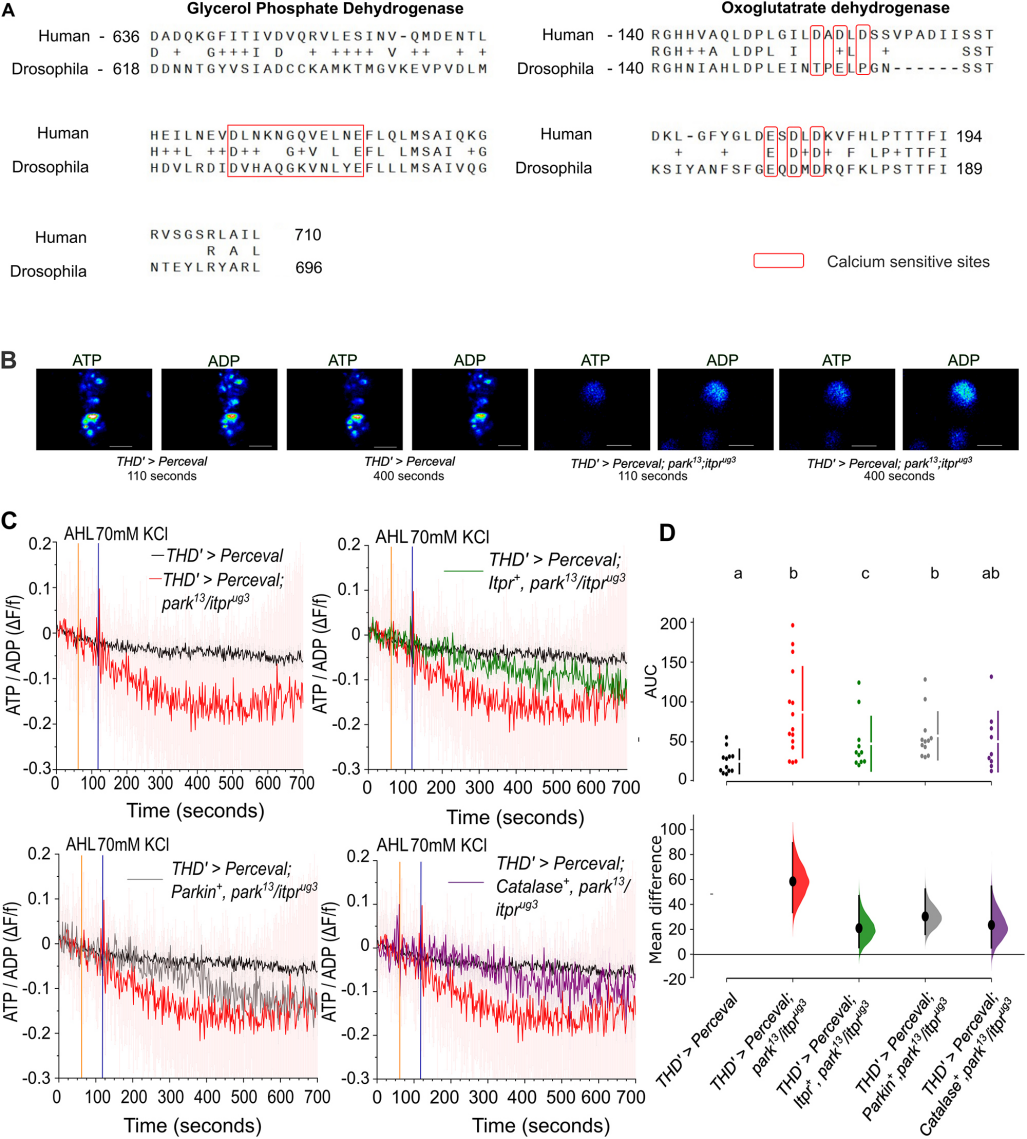
**Restoration of the ATP/ADP ratio post depolarisation is attenuated in PPL1 neurons of *park^13^/itpr^ug3^* animals.** (A) Pairwise alignment of Ca^2+^-sensitive enzymes between *Drosophila melanogaster* and *Homo sapiens*. Glycerol phosphate dehydrogenase protein sequence from human (region 636-710) and *Drosophila* (region 618-696) aligned using Smith–Waterman algorithm (left). BLOSUM62 matrix, similarity 44/70 (62.86%), identity 23/70 (32.86%). Oxoglutarate dehydrogenase protein sequence from human (region 140-194) and *Drosophila* (region 140-189) aligned using the Smith–Waterman algorithm (right). The marked boxes represent known regions of Ca^2+^ sensitivity being conserved in both organisms. BLOSUM62 matrix, similarity 32/56 (57.14%), identity 23/56 (41.07%). (B) Representative confocal images of PPL1 neurons for visualisation of ATP (488 nm) and ADP (405 nm) before (110 s) and after (400 s) addition of KCl (70 mM) in the indicated genotypes using the PercevalHR fluorescent sensor. Images were acquired at 2 FPS. Scale bars: 5 μm. (C) Traces show the normalised ratio of ATP/ADP as measured by fluorescent changes in Perceval at 405 nm (ADP) and 488 nm (ATP) after addition of adult haemolymph-like saline (AHL; 60 s) followed by KCl (70 mM, 120 s) in the indicated genotypes. WT flies expressing Perceval biosensor in a subset of DA neurons, *THD′>Perceval* (black); the heteroallelic mutant *THD′>Perceval; park^13^/itpr^ug3^* (red); overexpression of *itpr* (dark green), *parkin* (grey) and *Catalase* (violet) in the background of *THD′>Perceval; park^13^/itpr^ug3^*. (D) AUC of the traces shown in B quantified from 150 s to 700 s. Individual points represent the AUC of a single PPL1 neuron. The lower panel shows the effect size compared to the control genotype, *THD′>Perceval*. *THD′>Perceval* (black; *N*=8, *n*=12), heteroallelic mutant *THD′>Perceval; park^13^/itpr^ug3^* (red; *N*=5, *n*=15), overexpressed *THD′>Perceval; Itpr^+^, park^13^/itpr^ug3^* (green; *N*=6, *n*=11), *THD′>Perceval; Parkin^+^, park^13^/itpr^ug3^* (grey; *N*=6, *n*=12) and *THD′>Perceval; Catalase^+^, park^13^/itpr^ug3^* (green; *N*=6, *n*=9). *P*<0.05, as judged by Mann–Whitney test. Individual comparisons of genotypes with their *P*-values are provided in Table S11. Different lower-case letters represent significant differences from AUCs of the individual traces of other genotypes.

Mitochondrial ROS, which are converted to H_2_O_2_, are a by-product of the electron transport chain, required for generating ATP, which is, in turn, essential for neuronal firing (Sugioka et al., 1988; Zampese et al., 2022). The presence of excess H_2_O_2_ in the PPL1 neurons of *park^13^/itpr^ug3^* flies suggested inefficient ATP production leading to mitochondrial stress, previously reported in *Drosophila* Parkin mutants (Vincow et al., 2013; Whitworth et al., 2005) but not in *Drosophila* IP_3_R mutants. A reported cause of mitochondrial stress upon changes in Ca^2+^ signalling is the requirement for Ca^2+^ as a co-factor for ATP-generating enzymes in mammalian mitochondria. These include FAD-dependent glycerol phosphate dehydrogenase on the inner mitochondrial membrane, which catalyses the reverse reaction of glycerol-3-phosphate (G3P) back to dihydroxyacetone phosphate (DHAP) (Hansford and Chappell, 1967); oxoglutarate dehydrogenase, a component of the tricarboxylic acid cycle located on the mitochondrial matrix (McCormack and Denton, 1979); isocitrate dehydrogenase, localised in the cytosol, mitochondria and peroxisomes and involved in the decarboxylation of isocitrate (Denton et al., 1978); and pyruvate dehydrogenase, located on the mitochondrial matrix and required for decarboxylating pyruvate (Denton et al., 1972). The conservation of Ca^2+^-binding sites in the EF hand domains of FAD-dependent glycerol phosphate dehydrogenase (Denton, 2009; Lehn et al., 1994; Novials et al., 1997) and oxoglutarate dehydrogenase (Nagase et al., 2001; Zhong et al., 2022) between human and fly enzymes (Fig. 7A) indicates that fly neurons, with high ATP demand, undergo mitochondrial stress in the absence of optimal ER-mitochondrial Ca^2+^ transfer.

The ability of PPL1 neurons from *park^13^/itpr^ug3^* flies to convert ADP to ATP upon receiving a depolarisation stimulus (KCl) was tested next using a dual excitation/single emission fluorescent sensor, PercevalHR (Morris et al., 2020). The conversion of ADP to ATP showed a significant reduction upon depolarisation in mitochondria of PPL1 neurons from *park^13^/itpr^ug3^* flies compared to those from heterozygotes or control flies (Fig. 7B,C; Fig. S6). Overexpression of *Itpr^+^* resulted in partial rescue of the change in ADP to ATP, as did overexpression of *Catalase^+^*, although to a lesser extent, but there was no rescue by overexpression of *Parkin^+^* (Fig. 7D). These data suggest that the excess H_2_O_2_ generated in the dopaminergic neurons of *park^13^/itpr^ug3^* flies, while supporting supply of electrons to the electron transport chain, also becomes detrimental to mitochondrial and cellular health (Burbulla et al., 2017).

## DISCUSSION

In this study, we have identified a strong interaction between the genes encoding Parkin and IP_3_R in the context of dopaminergic neuron function. Interestingly, the key cellular pathways affected by *parkin* mutants (mitophagy) and *itpr* mutants (ER-Ca^2+^ release and mitochondrial Ca^2+^ entry) exhibit only minor deficits. We observed a slightly higher level of basal mitophagy in *park^13^/itpr^ug3^* mutant neurons than in control neurons, coupled with normal ER-Ca^2+^ release, and mitochondrial Ca^2+^ entry in a majority of the *park^13^/itpr^ug3^* mutant neurons. In agreement with a recent paper demonstrating that Parkin and PINK1 regulate ER-Ca^2+^ release through the IP_3_R (Ham et al., 2023), we did observe a few neurons that exhibit higher ER-Ca^2+^ release and mitochondrial Ca^2+^ entry signals in the *park^13^/itpr^ug3^* animals than in control animals (Fig. 3). Thus, the individual single-copy mutations of *park^13^* and *itpr^ug3^* have a minimal impact on the phenotypes associated with the two mutant pathways. Instead, the minor deficits in each pathway synergise and have a negative impact on mitochondrial energy production upon neuronal depolarisation (Fig. 7). Higher DOPAC levels observed in *park^13^/itpr^ug3^* animals (Fig. 6) suggest that dopamine oxidation is one alternative source for overcoming this energy deficit, with H_2_O_2_ as a by-product (Graves et al., 2020). Another source of excess H_2_O_2_ is presumably an overactive electron transport chain (Miwa et al., 2003). Absence of flight rescue by overexpression of Sod2 (Fig. 5A), supports the idea that excess H_2_O_2_ is generated as a compensatory mechanism. Although H_2_O_2_ feeds electrons to the electron transport chain, our data suggest that the process is inefficient, and cells using this mechanism end up with toxic levels of H_2_O_2_ and multiple levels of oxidative damage. The rescue by overexpression of Catalase suggests that excess mitochondrial H_2_O_2_ diffuses into the cytosol, as observed previously under conditions of mitochondrial stress (Koren et al., 2023). Further, a recent publication supports direct transfer of H_2_O_2_ from mitochondria to Catalase-containing peroxisomes (DiGiovanni et al., 2025). Thus, overexpression of *Catalase^+^* reverses the toxicity and improves neuronal function (Fig. 5; Fig. S4) by improving ATP/ADP ratios (Fig. 7). We also observed better rescues of excess H_2_O_2_ and the ATP/ADP ratio in PPL1 neurons with overexpression of *Itpr^+^* than in those with overexpression of *Parkin^+^* for reasons that are not clear.

The importance of mitigating oxidative stress in dopaminergic neurons is further emphasised by a study in which feeding an antioxidant, resveratrol, reversed the motor deficits of Parkin mutant flies (Adedara et al., 2022). Taken together, our results identify oxidative stress as an early contributor to malfunction of dopaminergic neurons in PD, and suggest that mitophagy and other neurodegenerative phenotypes arise at later stages after oxidative stress remains uncontrolled. These findings suggest that genetic or environmental factors, that together add to oxidative stress of dopaminergic neurons, are likely to contribute to cases of sporadic PD.

## MATERIALS AND METHODS

### Fly strains

All fly strains were reared on a diet consisting of 80 g corn flour, 20 g glucose, 40 g sugar, 15 g yeast extract, 4 ml propionic acid, 5 ml p-hydroxybenzoic acid methyl ester in ethanol and 5 ml ortho butyric acid in 1 litre. Flies were maintained at 25°C, unless otherwise mentioned under a 12:12 h light: dark cycle. The fly lines used include *CS*, *THGAL4* (Serge Birman, Centre National de la Recherche Scientifique, ESPCI Paris Tech, Paris, France), *THD'GAL4* [Mark N. Wu, Johns Hopkins University, Baltimore, MD, USA; Bloomington *Drosophila* Stock Center (BDSC) 93704], *UAS Perceval* (obtained from Knoblich laboratory, Institute of Molecular Biotechnology, Vienna, Austria), *UAS GCaMP6m* (BDSC 42748), *UAS MitoGCaMP5* (obtained from Dr Fumiko Kawasaki, University of Tokyo, Tokyo, Japan), *UAS SPLICS* (BDSC 95119), *nSybGAL4* (BDSC 5136), *DmefGal4* (BDSC 27390), *UAS Parkin^+^* (BDSC 95260), *UAS Catalase^+^* (BDSC 24621), *UAS Itpr^+^* (Venkatesh et al., 2001; BDSC 30742), *itpr^ka901^*, *itpr^ka1091^*, *itpr^wc703^*, *itpr^ug3^*, *itpr^sv35^* (Joshi et al., 2004), *park^13^* (BDSC 79210), *park^1^* (BDSC 34747), *park^25^* (BDSC), *UAS Mito-roGFP2-Orp1* (BDSC 67673), *Parkin^IR-37509^* (BDSC 37509), *Parkin^IR-KK104383^* [Vienna *Drosophila* Resource Center (VDRC) 104363], *Parkin^IR-31259^* (BDSC 31259) (Perkins et al., 2015), *PINK1^B9^* (BDSC 34749).

### Flight assays

Flight assays were conducted as described by Manjila and Hasan (2018). Briefly, flies were collected on the required days (5, 15 and 25) and tested in batches of five to ten by placing the vial containing the flies on ice to anesthetise for 2-3 min. Flies were tethered carefully between the head and the thorax to a thin metal wire with a drop of nail polish. A small air puff was given to stimulate flight, and the flight duration was recorded manually for up to 15 min. For all controls of *GAL4* and *UAS* lines, the parental lines were crossed with *CS.* The flight data are represented using the online tool estimationstats.com (Ho et al., 2019) for depiction of observed values of flight times with their effect sizes and the deviation from the mean in comparison to the genetic controls.

### Staging

Larval-staging experiments were performed for the required genotypes as described previously (Joshi et al., 2004). The number of viable organisms and the developmental stage were scored at specific developmental time points as mentioned below and in figure legends.

Synchronised egg laying was allowed for a 6-h period at 25°C for the mentioned genotypes. At 80-86 h after egg laying (AEL), batches of 25 heteroallelic mutant larvae were picked up by selecting against the dominant marker *Tubby* and transferred to a vial containing fresh media. Larval survival was observed at specified developmental time points (128-134 h and 176-182 h AEL), and the stage of development was determined along with the number of survivors. For each genotype, a minimum of three batches of 25 larvae were screened along with their control genotypes.

### Immunohistochemistry for assessment of mitochondrial morphology and ER-mitochondrial contacts, and counting of dopaminergic neurons

Adult fly brains were dissected in ice-cold 1× PBS and fixed in 4% paraformaldehyde in 1× PBS by shaking for 20 min at room temperature. Multiple washings (three to four times) were carried out with PBTx (0.3% Triton X-100 in 1× PBS) at 10 min intervals after fixation, followed by blocking with 5% normal goat serum (NGS) in PBTx for 2 h at room temperature. The samples were incubated overnight at 4°C with primary antibodies diluted in 5% NGS+PBTx at the appropriate concentration, washed three times with PBTx, then incubated with the secondary antibody for 2 h at room temperature. Brains were mounted in Vectashield anti-fade mounting medium (Vector Laboratories, SKU:H-1000-10). Confocal images were acquired using either an Olympus FV3000 or LSM 980 Airyscan and processed with ImageJ software.

Primary antibodies used were as follows: chick anti-GFP (1:8000; Abcam, ab13970; RRID: AB_300798) and mouse anti-TH (1:50; Immunostar, 22941; RRID: AB_572268). The secondary antibodies used were goat anti-chicken IgY (H+L), Alexa Fluor 488 (Thermo Fisher Scientific, A-11039; RRID: AB_2534096), goat anti-mouse IgG (H+L) Alexa Fluor 568 (Invitrogen, A-11004; RRID: AB_2534072).

For measuring ER-mitochondrial contact sites, brains were dissected in ice-cold adult haemolymph saline from the appropriate strains expressing the split GFP strain *UAS SPLICS*, in which GFP fluorescence is reconstituted when the ER and mitochondrial are at a distance of 8-10 nM. Brains were fixed in 4% paraformaldehyde for 20 min at room temperature. Post-fixation, they were imaged immediately using an Olympus FV3000 at 60× magnification. Proximal neurites were imaged close to the cell body. SPLICS punctae in green were counted from complete *z*-stacks and an area of 50 µm.

### *Ex vivo* live imaging of cytosolic GCaMP and MitoGCaMP

*Ex vivo* live imaging was conducted according to the protocol outlined by Sharma and Hasan (2020). On the designated day, adult brains were dissected in adult haemolymph-like saline (AHL; 2 mM CaCl_2_, 5 mM KCl_2_, 5 mM HEPES, 8.2 mM MgCl_2_, 108 mM NaCl, 4 mM NaHCO_3_, 1 mM NaH_2_PO_4_, 10 mM sucrose, 5 mM trehalose at pH 7.5). Dissected brains were placed on a 35 mm punched dish with a coverslip adhered to the bottom and embedded in ∼6 µl of 0.8-1% ultrapure low-melt agarose and bathed in AHL.

Time-series imaging was performed on an Olympus FV3000 inverted microscope using an *xy* plane and a 20× oil objective. Cytosolic and mitochondrial Ca^2+^ levels were monitored using GCaMP and MitoGCaMP, respectively, under 488 nm excitation. Ca^2+^ dynamics were monitored by measuring changes in fluorescence intensity. Stimulation was induced using the FMRFa peptide (DPKQDFMRFa, NeoBioLab), with a final concentration of 5 μM in a total volume of 100 μl. For GCaMP, AHL was added at the tenth frame, followed by FMRFa at the 40th frame, with images captured at a rate of one frame per second. For MitoGCaMP, AHL was added at the 30th frame and FMRFa at the 100th frame, with images taken every 1.5 s.

Raw fluorescence data were extracted by marking regions of interest (ROIs) using the time series analyzer plugin. ΔF/f was computed for each time point (t) using the formula ΔF/f=(F_t_−F_0_)/F_0_, where F_0_ represents the average basal fluorescence of the initial 30 frames.

Cells exhibiting the most robust responses among the visualised cells were selected for further analysis. To quantify the response to stimuli, the area under the curve (AUC) was calculated using OriginPro Learning Edition software (MicroCal, Origin Lab). The specific time frame for AUC calculations is provided in the figure legends.

### Mitophagy analysis with MitoQC

Flies of the designated genotypes were reared on cornmeal agar medium that was either supplemented with 5% sucrose solution or 20 mM paraquat resuspended in 5% sucrose. Upon reaching the required age, the adult brains were dissected in AHL and fixed in 4% paraformaldehyde for 20 min, followed by washing with ice-cold PBS. The brains were mounted in 70% glycerol for immediate analysis. Images were taken using the 60× oil objective of the Olympus FV3000 inverted microscope using an *xy* plane and were *z*-stacked to obtain the final images using the 405 nm and 488 nm excitation lines for capture of the MitoQC sensor. The analysis was carried out by installing the MitoQC plugin to the ImageJ software as mentioned in Montava-Garriga et al. (2020). For the selected ROIs, a ratio of mCherry to GFP was generated, and the number of mitolysosomes was counted using the MitoQC plugin.

### Measurement of mitochondrial H_2_O_2_

On the designated day, adult brains of the appropriate genotypes were dissected in cold 1× PBS and fixed in 4% paraformaldehyde in 1× PBS for 25 min. After fixation, the specimens were washed in cold 1× PBS and mounted in 70% glycerol. Notably, all solutions employed during the procedure were supplemented with 2 mM N-ethyl maleimide to mitigate the oxidation of H_2_O_2_. Images were obtained using the 60× oil objective of the Olympus FV3000 inverted microscope using an *xy* plane and were *z*-stacked to obtain the final image. *Mito-GFP2-Orp1* imaging was performed at 405 nm and 488 nm as described (Barata and Dick, 2013)*.* The raw data containing the area (A), mean grey value (MGV) and integrated density (ID) were extracted, and the corrected total cell fluorescence (CTCF) was calculated using the formula CTCF=ID of the ROI−(A*MGV).

### Liquid chromatography-tandem mass spectrometry analysis of *Drosophila* whole-brain dopaminergic and DOPAC metabolites

#### Sample preparation for mass spectrometry

Adult fly brains of the required genotypes and age were dissected and collected in a microcentrifuge vial in groups of 60, followed by suspension in liquid nitrogen for ∼20 s. The brains were homogenised in 80 µl of 0.1% formic acid containing 15.625 ng/ml phenyl alanine as an internal standard. Centrifugation was carried out at 1000 ***g*** for 10 min at 4°C. The collected supernatant was then mixed with three parts of acetonitrile, followed by dry heating at 70°C for 3 min and then incubation at 4°C on a vertical rocker for 60 min. An additional centrifugation step was carried out at 15,500 ***g*** at 4°C for 10 min. Supernatant was subjected to speed vacuum for 90 min and reconstituted in 0.1% formic acid with 10 mM aceto formide.

#### Preparation of calibration curves for mass spectrometry

A 16-point calibration curve was constructed using serial dilutions of the reconstituted standards of dopamine and DOPAC in 10 mM ammonium acetate buffer containing 0.1% formic acid, with the highest concentration as 1000 ng/ml and the lowest as 0.035 ng/ml. Phenylalanine internal standard (15.625 ng/ml) was added to all calibration points, followed by vortexing and centrifugation. Lastly, 10 µl of each sample was injected for mass spectrometric analysis.

#### Liquid chromatography conditions

A Phenomenex (C18), Synergi 4u, Fusion-RP-80A, 150 mm×4.6 mm column was used on an Agilent 1290 Infinity II instrument. Mobile phase A consisted of 10 mM ammonium acetate in water with 0.1% formic acid, and mobile phase B consisted of methanol with 0.1% formic acid. The column flow rate was adjusted to 0.5 ml/min and the column oven to 45°C. The auto sampler was maintained at 4°C. The run time was 20 min with the following gradient: 0-3 min at 2% B, 3-12 min at 35% B, 12-16 min at 90% B, 10 min at 90-5% B, 10-20 min at 5% B.

#### Mass spectrometry conditions

The instrument used was an Agilent 6495 Triplequad. The acquisition type was multiple reaction monitoring, positive and negative mode.

### Homology alignment

To assess the homology between two enzyme sequences, a pairwise alignment tool was utilised from the SnapGene (SnapGene Software, 2023) (version 7.2.1) software that uses the Smith–Waterman algorithm and a BLOSUM62 matrix. The resulting alignments were visually inspected to identify regions with conserved residues as previously identified from the references.

### Measuring the ATP to ADP ratio

Live imaging was employed to measure the changes in the ATP/ADP ratio in neurons using PercevalHR (Morris et al., 2020), a dual excitation/single emission sensor. Relative emission at 520 nm by excitation at either 405 or 488 nm acts as a proxy for the ATP/ADP ratio. On the required day of analysis, brains were dissected as in the *ex vivo* live imaging protocol described above. Imaging was performed on an Olympus FV3000 inverted microscope using an *xy* plane and a 20× oil objective according to the time series shown in the figures. Fluorescence was captured along both the excitation lines of 405 nm and 488 nm. At the 30th frame, 7 μl AHL was added, followed by addition of 7 μl of 1 M KCl at the 60th frame so that the final concentration was maintained at 70 mM. Images were acquired at a rate of one frame every 2 s. Raw fluorescence data were extracted by marking ROIs using the time series analyzer plugin. ΔF/f was computed for each time point (t) using the formula ΔF/f=(F_t_−F_0_)/F_0_, where F_0_ represents the average basal fluorescence of the initial 30 frames.

Cells exhibiting the most robust responses among the visualised cells were selected for further analysis. To quantify the response to stimuli, the AUC was calculated using OriginPro Learning Edition software (OriginLab Corporation, 2024). The specific time frame for AUC calculation is described in the figure legends.

### Statistical analysis

Distributions not following the normal were subjected to non-parametric tests such as the Mann–Whitney *U*-test for significance, performed using the OriginPro Learning Edition software (OriginLab Corporation, 2024; MicroCal). Normal distributions were subjected to Student’s *t*-test or ANOVA (unequal variance) in Microsoft Excel for analysis of significance. All *P*-values have been enlisted in the figure legends or in Tables S1-S8, S10 and S11.

## Supplementary Material

10.1242/dmm.052146_sup1 Supplementary information
