# Supplementary material for: Synergism of IP3R and Parkin mutants identifies mitochondrial stress as an early feature of Parkinson's disease
Source: Dis Model Mech. 2026 Jan 21;19(1):dmm052146. doi: 10.1242/dmm.052146 (PMC12869514; doi:10.1242/dmm.052146)
Supplement: Supplementary information [file dmm-19-052146-s1.pdf]

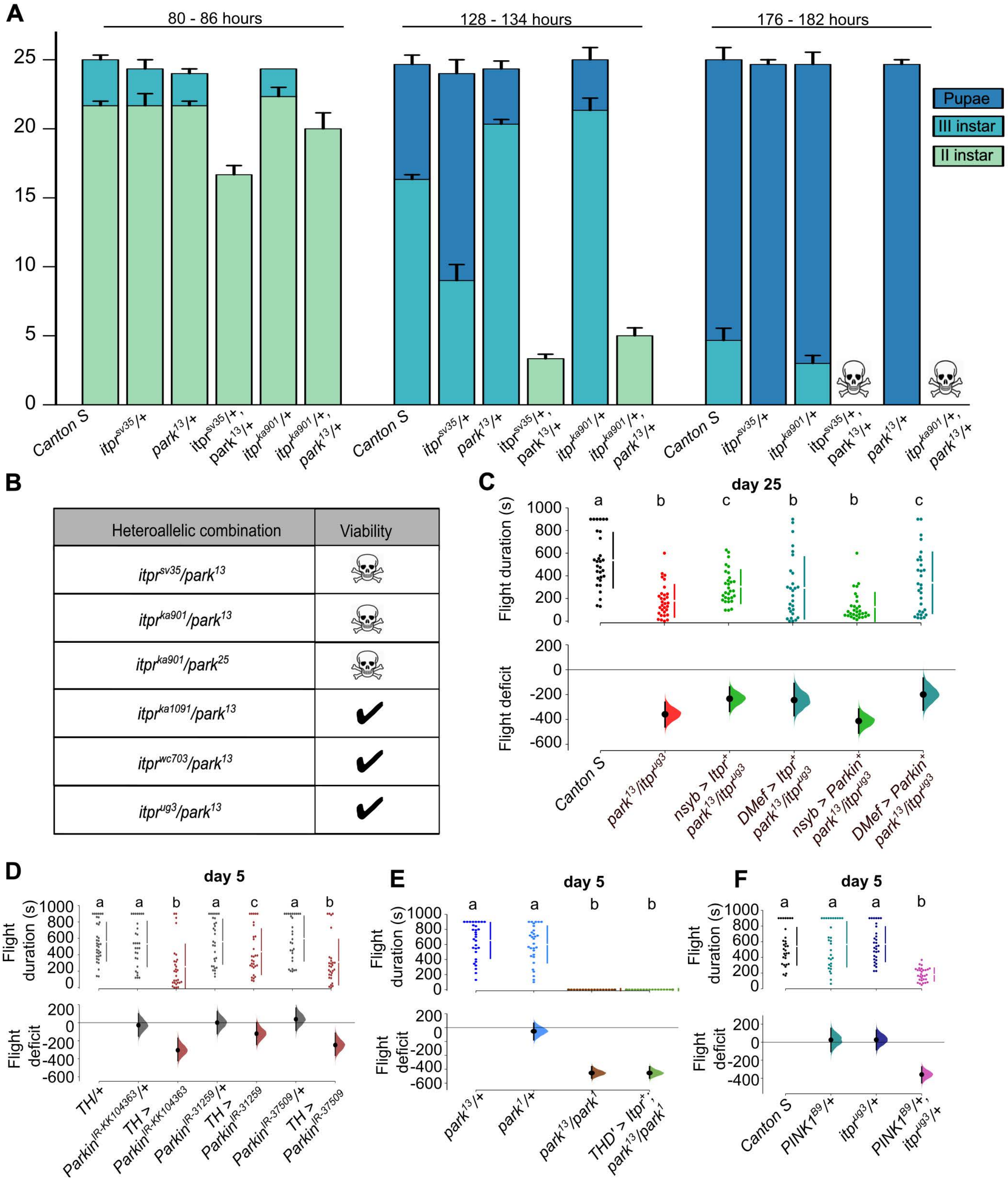

Fig. S1. Genetic interaction between Parkin and IP$_3$R mutants.

A. Larvae with one copy of the *park*^{13} (null) allele and one copy of either *itpr*^{sv35} (null) allele or *itpr*^{ka901}, a strong mutant allele, are lethal. The stack bar graph represents the number of viable second instar larvae (blue-green), third instar larvae (coral blue) and pupae (sea blue) of the indicated genotypes at the indicated time after egg laying

(AEL). Viability of heterozygotes of *park*^{13}/+, *itpr*^{sv35}/+ *and itpr*^{ka901}/+ is similar to wild type (*Canton S*) larvae. N=3 and n=25 where N is number of experiments and n is number of larvae in each experiment. For 80-86 hours II instar larvae, the (mean ± SEM) values include *Canton S* (21.6667 ± 0.3333), *park*^{13}/+ (21.6667 ± 0.8819), *itpr*^{sv35}/+ (21.6667 ± 0.3333), *itpr*^{sv35}*/park*^{13} (16.6667 ± 0.6667), *itpr*^{ka901}/+ (22.3333 ± 0.6667), *ka*^{901}*/park*^{13} (20 ± 1.1547). For 80-86 h III instar larvae, the (mean ± SEM) values include *Canton S* (3.3333 ± 0.3333), *park*^{13}/+ (2.6667 ± 0.6667), *itpr*^{sv35}/+ (2.3333 ± 0.3333), *itpr*^{ka901}/+ (2 ± 0). For 128-136 hours II instar larvae, the (mean ± SEM) values include *itpr*^{sv35}*/park*^{13} (3.3333 ± 0.3333), *itpr*^{ka901}*/park*^{13} (5 ± 0.5773). For 128-136 h III instar larvae, the (mean ± SEM) values include *Canton S* (16.3333 ± 0.33333), *park*^{13}/+ (15 ± 1), *itpr*^{sv35}/+ (20.3333 ± 0.33333), *itpr*^{ka901}/+ (21.33333, 0.8819). For 128-136 h Pupae, the (mean ± SEM) values include *Canton S* (8.3333 ± 0.6667), *park*^{13}/+ (9 ± 1.1547), *itpr*^{sv35}/+ (4 ± 0.5773), *itpr*^{ka901}/+ (3.6667 ± 0.8819). For 176-182 hours III instar larvae, the (mean ± SEM) values include *Canton S* (4.66667 ± 0.8819), *itpr*^{sv35}/+ (3 ± 0.5773). For 176-182 hours pupae, the (mean ± SEM) values include *Canton S* (20.3333 ± 0.8819), *park*^{13}/+ (24.66667 ± 0.3333), *itpr*^{sv35}/+ (21.66667 ± 0.8819), *itpr*^{ka901}/+ (24.6667 ± 0.3333). Individual comparisons of genotypes with their P values are given in Table S2. Test for significance was interpreted using Single factor ANOVA.

B. Identification of adult viable heterozygous combinations of *park*^{13} with *itpr* mutant alleles.

C. Rescue of flight deficits in *park*^{13}*/itpr*^{ug3}. Partial rescue of flight is observed upon pan-neuronal (*nsybGAL4*) overexpression of *Itpr*$^{+}$ but not upon overexpression of *Parkin*$^{+}$. In contrast flies with expression of Parkin+ in muscles (*Dmef2GAL4*) exhibit partial rescue of flight, whereas there was rescue of flight upon expression of *Itpr*$^{+}$ in

muscles. *Canton S* (N=30), *park*^{13}*/itpr*^{ug3} (N=31), the pan neuronal (*nsybGAL4*) expression of *Itpr* (N=30) and *Parkin* (N=30) (light green), and the flight muscle (*dmefGAL4*) expression of *Parkin* (N=30) and *Itpr* (N=28) (teal). Test for significance was interpreted using a non-parametric, Mann-Whitney test.

D. Parkin is required for maintaining flight bout durations in dopaminergic cells marked by *THGAL4*. Flight duration of flies with knockdown of *Parkin* in *THGAL4* marked cells using three different Parkin RNAi lines are shown with appropriate genetic controls. TH/+ in black (N=35) with different Parkin RNAi lines (all in brown) *Parkin*$^{IR\text{-}KK104363}$ (N=29), *Parkin*$^{IR\text{-}31259}$ (N=30), and *Parkin*$^{IR\text{-}37509}$ (N=29), Parkin RNAi controls (all in dark gray) *Parkin*$^{IR\text{-}KK104363}$/+ (N=29), *Parkin*$^{IR\text{-}37509}$/+ (N=29) for *Parkin*$^{IR\text{-}31259}$/+ (N=30). Test for significance was interpreted using a non-parametric, Mann-Whitney test.

E. The double mutant combination of *park*^{13}*/park*^{1} is flightless (brown) whereas normal flight bout durations are observed in heterozygous controls *park*^{13}/+ (royal blue) and *park*^{1}/+ (light blue). Overexpression of *Itpr*+ (olive) in flight promoting dopaminergic neurons (*THD'GAL4*) did not rescue flight. N=30 for all genotypes. Test for significance were interpreted using a non-parametric, Mann-Whitney test.

F. The double mutant combination of *PINK1*^{B9}*/itpr*^{ug3} exhibits a strong flight deficit (pink) whereas normal flight bout durations are observed in heterozygous controls *PINK1*^{B9} /+ (teal blue) and *itpr*^{ug3}/+ (dark blue). *Canton S* (N=31), *PINK1*^{B9} /+ (N=30), *itpr*^{ug3}/+ (N=31), *PINK1*^{B9}*/itpr*^{ug3} (N=30). Test for significance were interpreted using a non-parametric, Mann-Whitney test.

Flight durations in panels C – F are shown as swarm plots (top) and effect size (bottom) compared with the first control genotype. Different letters represent significant difference from flight times of other genotypes (P<0.05) as seen by the

Disease Models & Mechanisms • Supplementary information

Mann-Whitney test. Individual comparisons of genotypes with their P values are given in Table S3.

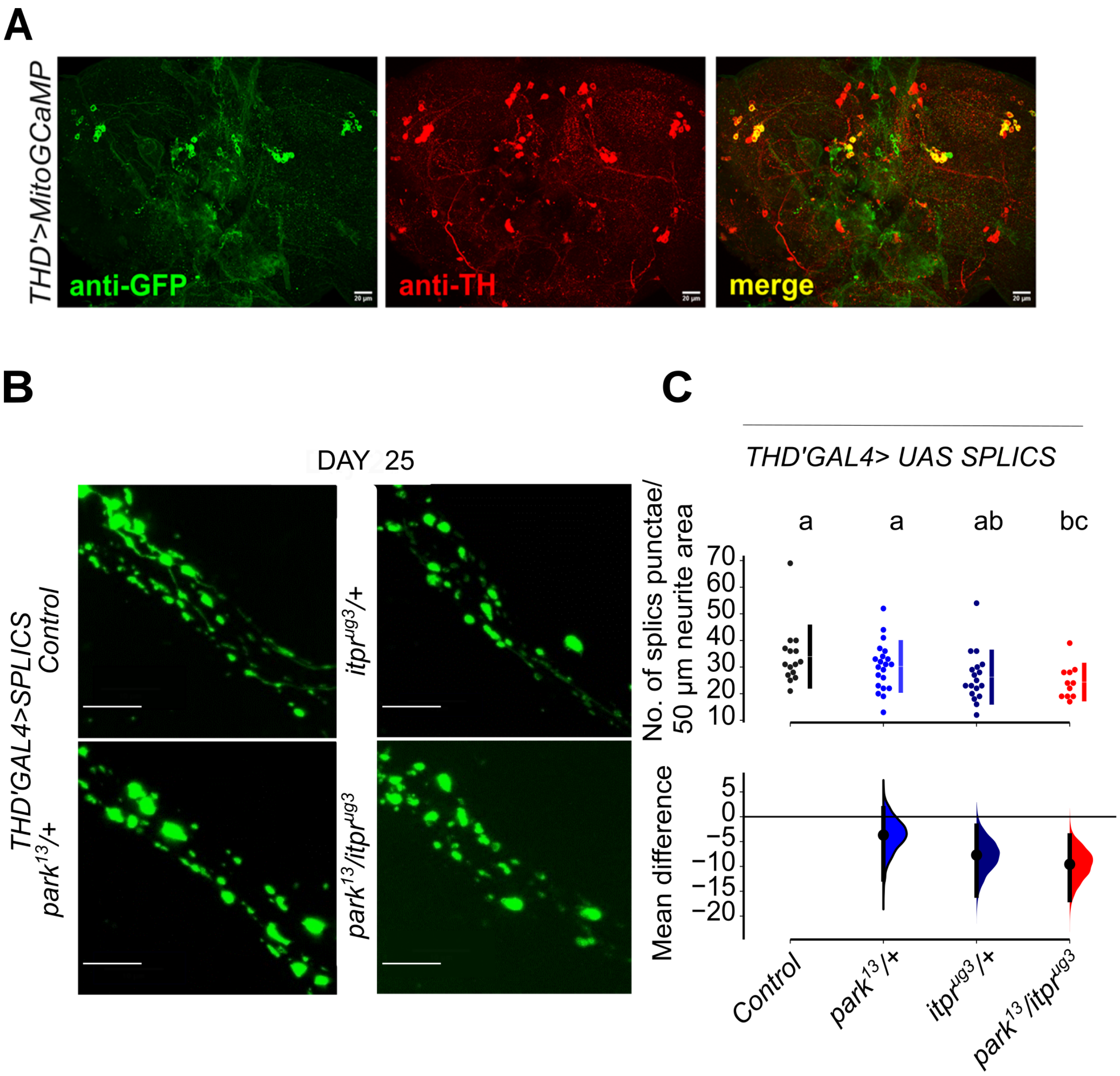

Fig. S2. Visualisation of brain dopaminergic neurons and ER-mitochondrial contact sites.

A. Confocal images of a *Drosophila* central brain marking the *THD' GAL4* expressing PPL1 and PPM3 clusters (anti-GFP, green), all TH positive cells visible in the imaged orientation (anti-TH, red) and a merged image. The scale bar represents 20 µm.

Disease Models & Mechanisms • Supplementary information

B. Representative images of ER-Mitochondrial contacts (8-10 nm) at day 25 in neurites from *THD'GAL4* dopaminergic neurons of the indicated genotypes. The scale bar represents 10 μm. A minimum of 5 brains were sampled for each genotype.

C. Quantification of ER-Mitochondrial contact punctae from a 50 μm square area in THD'GAL4 neurites. Different letters represent significant difference (P<0.05) as calculated by the Mann-Whitney test. $park^{13}$/+ is not significantly different from control. $itpr^{ug3}$/+ is significantly different from control but not from $park^{13}$/+. $park^{13}/itpr^{ug3}$ is significantly different from control and $park^{13}$/+ but not significantly different from $itpr^{ug3}$/+. Exact P values are in Table

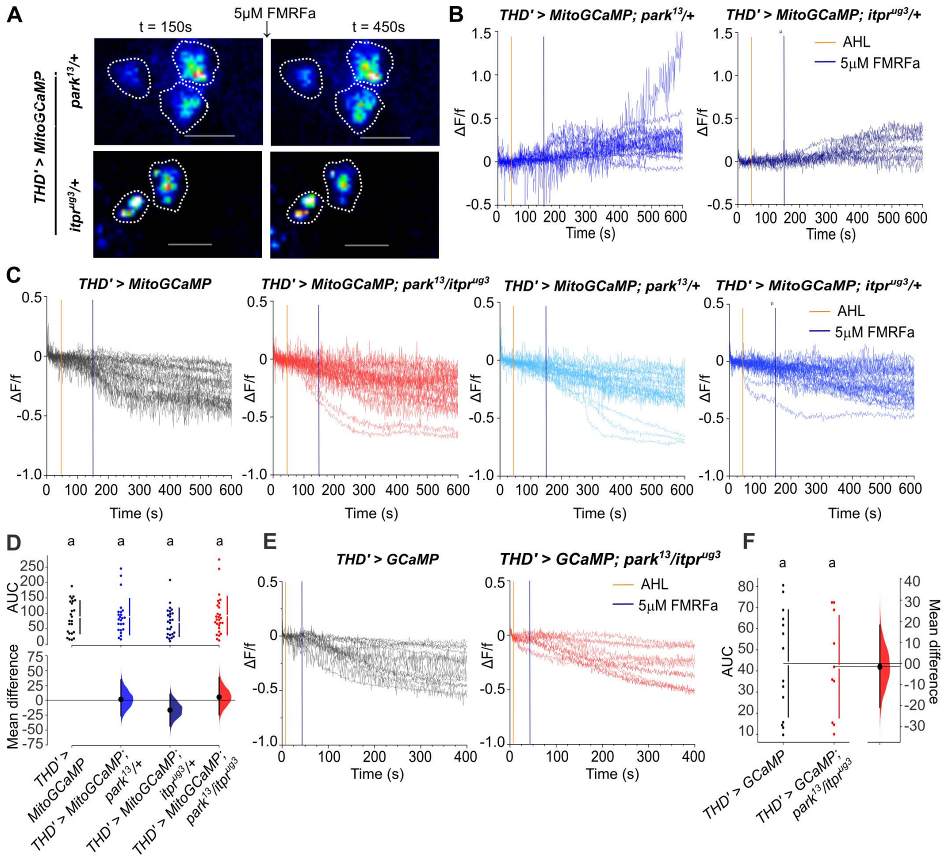

Fig. S3. Inhibitory traces of ER calcium release and mitochondrial uptake depict no change in *park*^{13}/*itpr*^{ug3} animals.

- **A.** Representative confocal image of the DA neurons (PPL1 cluster) expressing *mitoGCaMP3* to measure mitochondrial calcium before 5μM FMRFa addition at 150 seconds and 450 seconds of the indicated genotypes. Scale bars indicate 10 μm. Images were acquired at 1.5 FPS (left panel).
- **B.** Mitochondrial Ca^{2+} uptake (right panel) judged by MitoGCaMP3 between indicated genotypes (controls) in adult dopaminergic neurons. Individual traces of the genetic controls representing normalized changes in fluorescence to

MitoGCaMP3. The genetic controls of the heteroallelic mutant responding to changes in MitoGCaMP3, *park*^{13}/+ (N=7, n=19) in royal blue and *itpr*^{ug3}/+ in navy blue (N=6, n=11).

C. Traces representing inhibitory response to FMRFa in adult dopaminergic neurons of indicated genotypes judged by MitoGCaMP3. Individual traces of WT flies in black (N=6, n=16), the heteroallelic mutant in red (N=6, n=25) and the genetic controls of the heteroallelic mutant responding to changes in MitoGCaMP3, *park*^{13}/+ (N=7, n=24) in royal blue and *itpr*^{ug3}/+ in navy blue (N=6, n=25) representing normalized changes in fluorescence to MitoGCaMP3.

D. Comparison of inhibitory traces of the mitochondrial Ca^{2+} uptake depicts no change. Area under the curve (AUC) taken for each individual inhibitory trace of the mentioned genotypes for individual cells of dopaminergic neurons denoting mitochondrial Ca^{2+} uptake (right). The relative mean difference between the WT in black (no. of brains=5 and no. of cells=24), *park*^{13}/+ control in light blue (no. of brains=7 and no. of cells= 24), *itpr*^{ug3}/+ control in navy blue (no. of brains=6, no. of cells=27) and the heteroallelic mutant in red (no. of brains=6 and no. of cells=27) is non-significant as judged by the non-parametric Mann-Whitney test.

E. Traces representing inhibitory response to FMRFa in adult dopaminergic neurons of indicated genotypes judged by *UAS GCaMP6m*. Individual traces of WT flies in black (N=4, n=14), the heteroallelic mutant in red (N=5, n=12) representing normalized changes in fluorescence to GCAMP6m.

F. Comparison of inhibitory traces of the Ca^{2+} release from ER depicts no change. The Area under the curve taken for each individual trace of the mentioned genotypes for individual cells of dopaminergic neurons denoting ER Ca^{2+}

release (right). The relative mean difference between the WT in black (no. of brains=4 and no. of cells=15) and the heteroallelic mutant in red (no. of brains=4 and no. of cells=11) is non-significant as judged by unpaired two sample t-test (one tailed). Similar letters represent no significant difference between the genotypes. Individual comparisons of genotypes with their P values are given in Table S5.

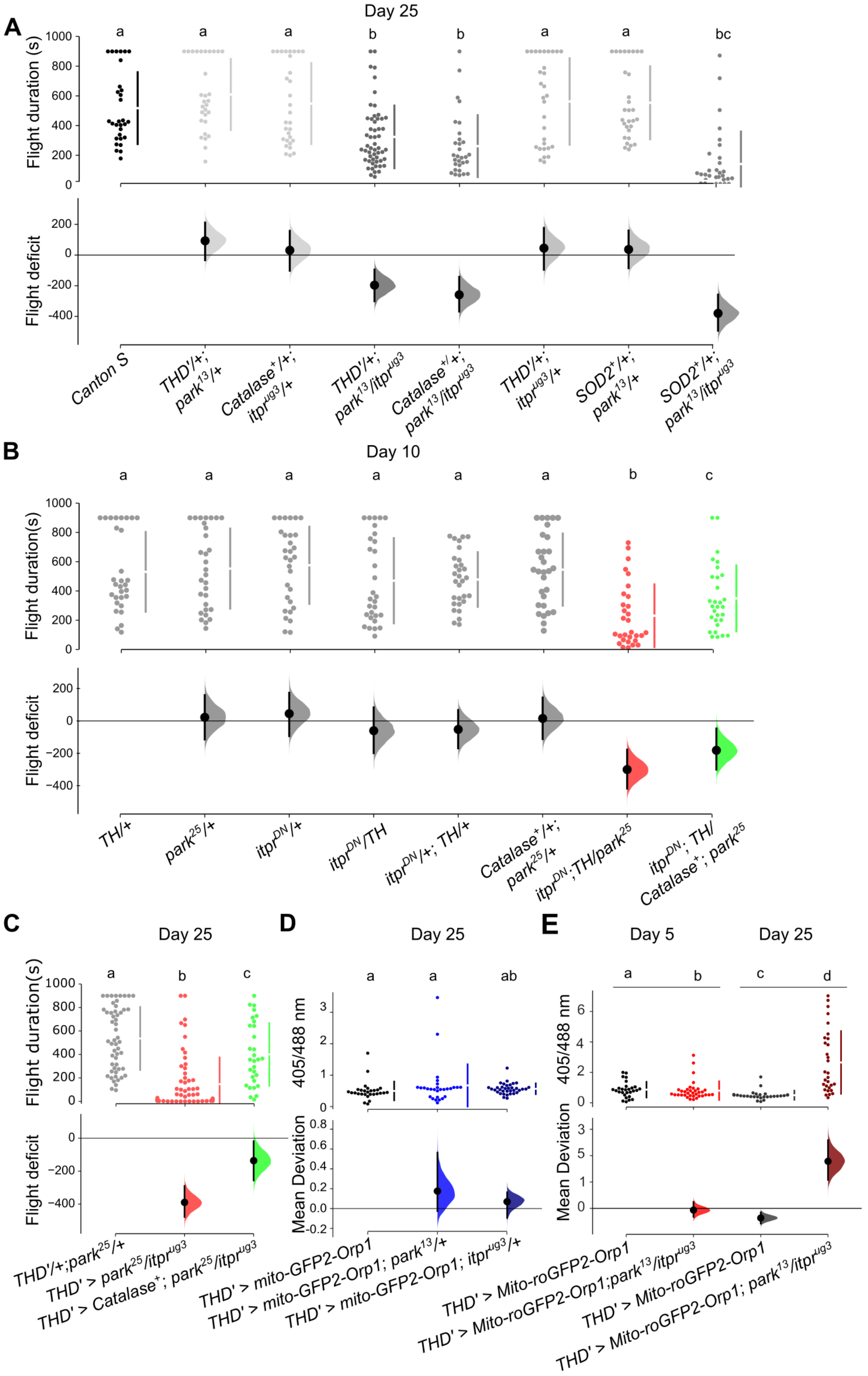
A
Day 25
Flight duration (s)
Flight deficit
Canton S
THD'/+; park13/+
Catalase+/+; itprug3/+
THD'/+; park13/itprug3
Catalase+/+; park13/itprug3
THD'/+; itprug3/+
SOD2+/+; park13/+
SOD2+/+; park13/itprug3
B
Day 10
Flight duration(s)
Flight deficit
TH/+
park25/+
itprDN/+
itprDN/TH
itprDN/+; TH/+
Catalase+/+; park25/+
itprDN;TH/park25
itprDN; TH/ Catalase+; park25
C
Day 25
Flight duration(s)
Flight deficit
THD'/+;park25/+
THD' > park25/itprug3
THD' > Catalase+; park25/itprug3
D
Day 25
405/488 nm
Mean Deviation
THD' > mito-GFP2-Orp1
THD' > mito-GFP2-Orp1; park13/+
THD' > mito-GFP2-Orp1; itprug3/+
E
Day 5
Day 25
405/488 nm
Mean Deviation
THD' > Mito-roGFP2-Orp1
THD' > Mito-roGFP2-Orp1;park13/itprug3
THD' > Mito-roGFP2-Orp1
THD' > Mito-roGFP2-Orp1; park13/itprug3

Fig. S4. Representation of flight data of control and mutant genotypes and comparison H_2O_2 data between 5 day and day 25 in *park^{13}/itpr^{ug3}* animals and their control genotypes.

A. Flight duration in the indicated control genotypes of 25d flies shown as swarm plots (top) and effect size (below). Each individual dot indicates the flight time of one fly. Genotypes were compared to WT flies (black) and amongst each other using the Mann-Whitney test. Different letters above each genotype indicate significant difference ($P<0.05$). Actual P values are given in Table S7. *Canton S* (N=30), *THD' GAL4; park^{13}/+* (N=30), *UAS Catalase*; *itpr^{ug3}/+,* (N=30),*THD' GAL4; park^{13}/itpr^{ug3}* (N=54), *UAS Catalase*; *park^{13}/itpr^{ug3}* (N=30), *THD'/+; itpr^{ug3}/+* (N=30), *UAS SOD2/+; park^{13}/+* (N=30)*,* and UAS SOD2/+; *park^{13}/itpr^{ug3}* (N=30).

B. Flight duration in the indicated control (grey), mutant (red) and rescue (green) genotypes of 10d flies shown as swarm plots (top) and effect size (below). Each individual dot indicates the flight time of one fly. Genotypes were compared to *THGAL4/+* flies (first grey plot) and amongst each other using the Mann-Whitney test. Different letters above each genotype indicate significant difference ($P<0.05$). Actual P values are given in Table S7. '+' indicates wildtype (*Canton S*). *TH GAL4/+* (N=30), *park^{25}/+*, (N=30), *itpr^{DN}/+* (N=30), *itpr^{DN}/TH GAL4* (N=30), *itpr^{DN}/+; TH GAL4/+* (single strain, N=30), *UAS Catalase^{+}/+; park^{25}/+* (N=30), *itpr^{DN}; TH GAL4/park^{25},* (N=30), *itpr^{DN}; TH GAL4/UAS Catalase^{+}; park^{25}* (N=30).

C. Flight duration in the indicated control (grey), mutant (red) and rescue (green) genotypes of 25d flies shown as swarm plots (top) and effect size (below). Each individual dot indicates the flight time of one fly. Genotypes were compared

Disease Models & Mechanisms • Supplementary information

amongst each other using the Mann-Whitney test. Heterozygotes of *park*^{25} and *itpr*^{ug3} (red, *THD'GAL4/+*; *park*^{25}*/itpr*^{ug3}, N=60) were compared to *THD'GAL4/+; park*^{25}*/+* flies (grey, N=53) and the rescue strain *THD' GAL4/UAS Catalase*$^{+}$*; park*^{25}*/itpr*^{ug3} (green, N=30) and found to be significantly different from both. The rescue strain (green) is significantly different from the mutant (red) and the control (grey). Different letters above each genotype indicate significant difference ($P<0.05$). Actual P values are given in Table S7.

D. Quantification of oxidized roGFP indicating H_2O_2 levels in PPL1 DA neurons at 5 Day and 25 Day of the indicated genotypes. Swarm plots demonstrating the ratio of fluorescence at 405nm to 488nm, in the mentioned genotypes, are shown on top. Each dot represents the ratio from a single cell (n). Different letters represent significant difference using the Mann-Whitney test. H_2O_2 levels appear elevated in *park*^{13}*/itpr*^{ug3} animals at 25 days in comparison with younger (5 day) flies. Genotypes at 5 days include *THD'GAL4>Mito-roGFP2-Orp1* in black (N=4, n=26), *THD'GAL4>Mito-roGFP2-Orp1*; *park*^{13}*/itpr*^{ug3} in red (N=6, n=47), and at 25 days include *THD'GAL4>Mito-roGFP2-Orp1* in dark grey (N=6, n=28), *THD'GAL4>Mito-roGFP2-Orp1*; *park*^{13}*/itpr*^{ug3} in maroon (N=7, n=30).

E. Quantification of oxidized roGFP indicating H_2O_2 levels in PPL1 DA neurons of control flies at 25 days. Genotypes include *THD'GAL4>Mito-roGFP2-Orp1* in black (N=6, n=28), *THD' > mito-roGFP2-Orp1*; *park*^{13} /+ royal blue (N=6, n=28), *THD' > mito-roGFP2-Orp1; itpr*^{ug3} /+ in navy blue (N=5, n=34). P-value < 0.05 as judged by Mann-Whitney test. Individual comparisons of genotypes with their P values are given in Table S7. Different letters represent significant difference from flight times and H_2O_2 levels of other genotypes.

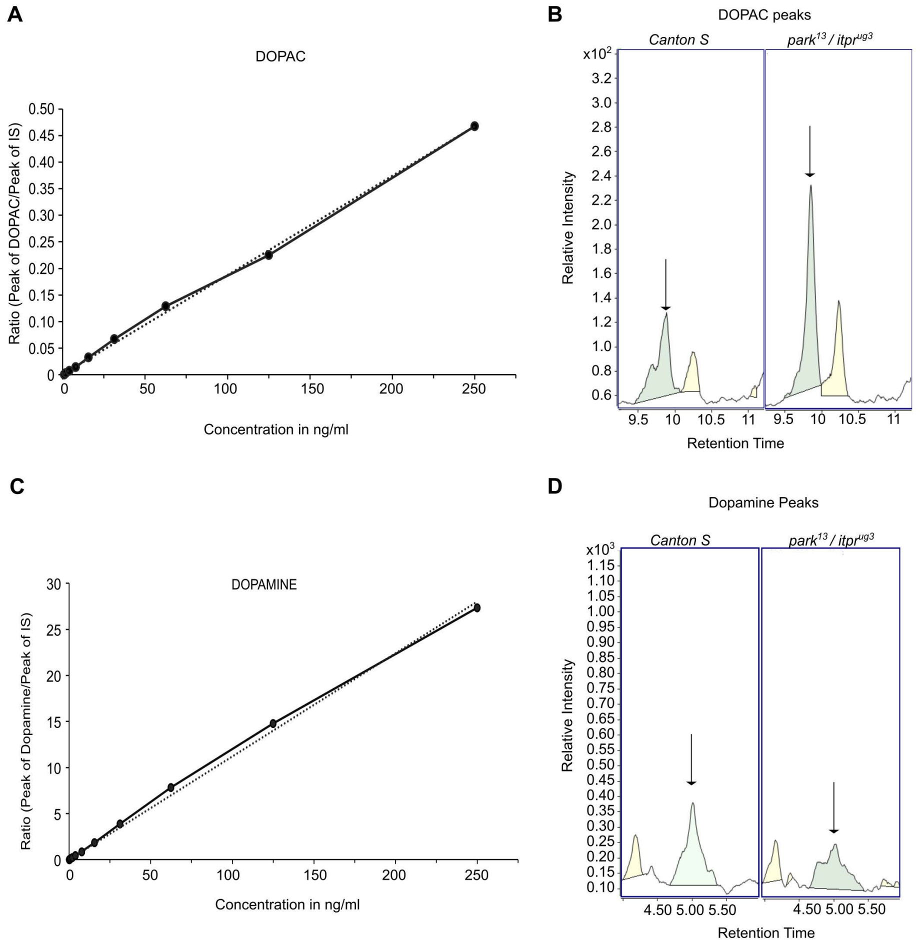

Fig. S5. Mass spectrometric analysis of DOPAC and dopamine from *Drosophila* heads.

A. Calibration data of DOPAC standards. Each point represents a particular concentration standard of DOPAC measured at the particular retention time of 10.2. and normalized against the value for the internal standard of Phenylalanine. R^2 = 0.999. The equation for the calibration graph is y=0.0019x after set intercept. The Limit of Detection (LOD) is 1.9531 ng/mL and the Limit of Quantitation (LOQ) is 3.9063 ng/mL

B. Individual peak of one set of DOPAC measurement of the mentioned genotype derived using mass spectrometry. The peaks measure relative intensity of the

Disease Models & Mechanisms • Supplementary information

compound at the particular retention time (RT - 9.8). The green shaded area was quantified (arrow).

C. Calibration data of Dopamine standards. Each point represents particular concentration standard of Dopamine measured at the particular retention time of 4.9) and normalized against the value for the internal standard of Phenylalanine. R^2 = 0.9982. The equation for the calibration graph is y=0.112x after set intercept. The Limit of Detection (LOD) is 0.2441 ng/mL and the Limit of Quantitation (LOQ) is 0.976 ng/mL

D. Individual peaks of one set of Dopamine derived using mass spectrometry. The peaks measure relative intensity of the compound at the particular retention time of 4.9. The green shaded area was quantified. Data for calibration along with the sample data of genotypes are given in Table S8.

Disease Models & Mechanisms • Supplementary information

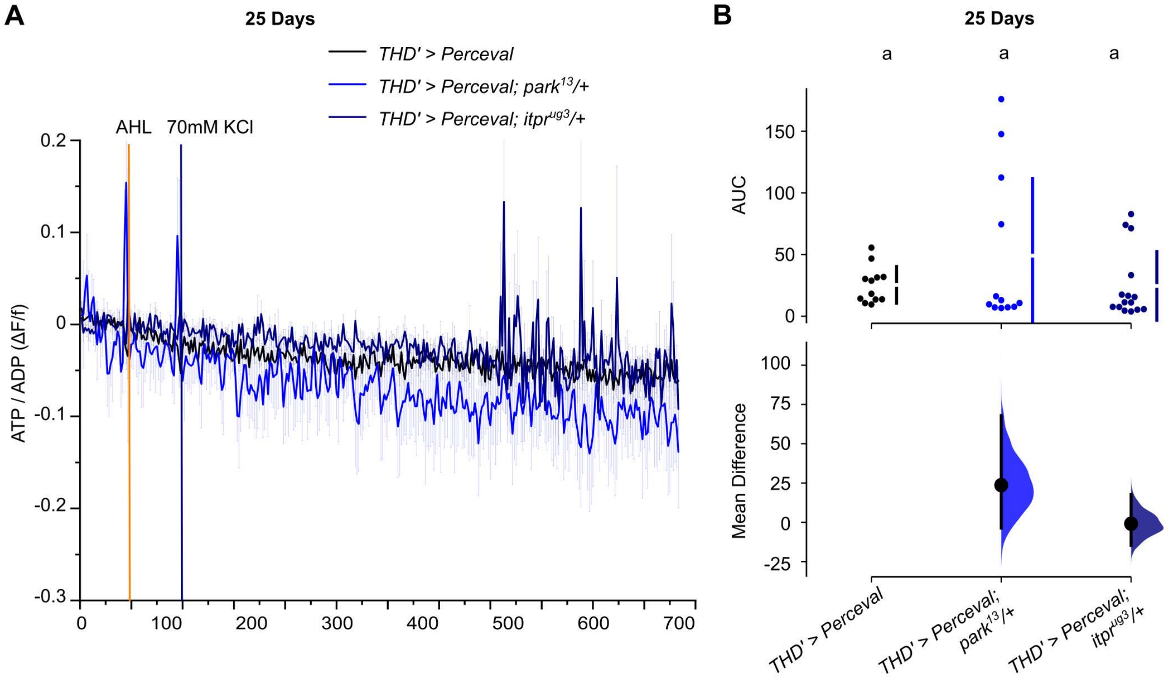

Fig. S6. Control genotypes measuring ATP/ADP ratios and their AUC quantification.

- **A.** Traces show the normalized ratio of ATP/ADP as measured by fluorescent changes in Perceval at 405 nm (ADP) and 488 nm (ATP) after addition of adult haemolymph-like saline (AHL, 60 s) followed by KCl (70 mM, 120 s) in the indicated genotypes of 25 Day old flies. Black represents the wild type control *THD' > Perceval*, and royal blue and navy blue represents genetic controls *THD' > Perceval*; *park*^{13} /+, and *THD' > Perceval; itpr*^{ug3} /+ respectively.
- **B.** Area under the curve (AUC) of each individual traces of the depicted genotypes A quantified from 150 seconds to 700 seconds. Individual points represent the AUC of a single PPL1 neuron. The lower panels show the effect size as compared to the wildtype control genotype *THD' > Perceval* (black (N=8, n=12)), and respective genetic controls *THD' > Perceval*; *park*^{13} /+ (royal blue, (N=5, n=12), *THD' > Perceval; itpr*^{ug3} /+ (navy blue, N=6, n=15).

  The exact P values are given in Table S10. Significance test – Non-parametric, Mann Whitney test for P-value. Different letters represent significant difference from AUC of the individual traces of other genotypes.

Disease Models & Mechanisms • Supplementary information

Table S1. P-values for Figure 1B-D

**Figure 1B**	**Test - Mann Whitney**		
**Genotype**	**Comparison**	**Day**	**P-value**
park^{13}/+	*Canton S*	5	0.157
itpr^{ug3}/+	*Canton S*	5	0.21445
park^{13}/*itpr*^{ug3}	*Canton S*	5	<0.0001
Canton S	*Canton S*	15	0.3 8
park^{13}/+	*Canton S*	15	0.02546
itpr^{ug3}/+	*Canton S*	15	0.07899
park^{13}/*itpr*^{ug3}	*Canton S*	15	<0.0001
Canton S	*Canton S*	25	0.00154
park^{13}/+	*Canton S*	25	0.00262
itpr^{ug3}/+	*Canton S*	25	<0.0001
park^{13}/*itpr*^{ug3}	*Canton S*	25	<0.0001
**Fig 1C**	**Test - Mann Whitney**		
**Genotype**	**Comparison**	**Day**	**p-value**
Canton S		25	~
park^{13}/*itpr*^{ug3}	*Canton S*	25	<0.0001
THD' > park^{13}/*itpr*^{ug3}	*Canton S*	25	<0.0001
UAS Parkin^{+} *> park*^{13}/*itpr*^{ug3}	*Canton S*	25	<0.0001
THD'> Parkin^{+}*; park*^{13}/*itpr*^{ug3}	*Canton S*	25	1.88E-04
THD'> Itpr^{+}*; park*^{13}/*itpr*^{ug3}	*Canton S*	25	0.06547
UAS Itpr^{+} *> park*^{13}/*itpr*^{ug3}	*Canton S*	25	<0.0001
THD' > park^{13}/*itpr*^{ug3}	*park*^{13}/*itpr*^{ug3}	25	0.00103
UAS Parkin^{+} *> park*^{13}/*itpr*^{ug3}	*park*^{13}/*itpr*^{ug3}	25	0.00318

Disease Models & Mechanisms • Supplementary information

THD'> Parkin^{+}*; park*^{13}*/itpr*^{ug3}	*park*^{13}*/itpr*^{ug3}	25	0.00987
THD'> Itpr^{+}*; park*^{13}*/itpr*^{ug3}	*park*^{13}*/itpr*^{ug3}	25	<0.0001
UAS Itpr^{+} *> park*^{13}*/itpr*^{ug3}	*park*^{13}*/itpr*^{ug3}	25	0.22768
THD'> Parkin+; park^{13}*/itpr*^{ug3}	*UAS Parkin*$^{+}$ *> park*^{13}*/itpr*^{ug3}	25	0.7449
THD'> Parkin+; park^{13}*/itpr*^{ug3}	*THD' > park*^{13}*/itpr*^{ug3}	25	0.8013
THD'> Itpr^{+}*; park*^{13}*/itpr*^{ug3}	*UAS Itpr*$^{+}$ *> park*^{13}*/itpr*^{ug3}	25	0.00717
THD'> Itpr^{+}*; park*^{13}*/itpr*^{ug3}	*THD' > park*^{13}*/itpr*^{ug3}	25	0.06051
**Fig 1D**	**Test - Mann Whitney**		
**Genotype**	**Comparison**	**Day**	**p-value**
Parkin$^{IR\text{-}37509}$*/+*	*THD'/+*	25	0.51916
THD'> Parkin$^{IR\text{-}37509}$	*THD'/+*	25	0.06966
THD'/+;itpr^{ug3} */+*	*THD'/+*	25	0.87672
THD';itpr^{ug3}*> Parkin*$^{IR\text{-}37509}$	*THD'/+*	25	<0.0001
Parkin$^{IR\text{-}37509}$*/+*	*THD'> Parkin*$^{IR\text{-}37509}$	25	0.00271
THD'/+;itpr^{ug3}*> Parkin*$^{IR\text{-}37509}$	*THD';itpr*^{ug3} */+*	25	<0.0001
THD/+';itpr^{ug3}*> Parkin*$^{IR\text{-}37509}$	*Parkin*$^{IR\text{-}37509}$*/+*	25	<0.0001
THD'/+;itpr^{ug3}*> Parkin*$^{IR\text{-}37509}$	*THD'> Parkin*$^{IR\text{-}37509}$	25	<0.0001

Disease Models & Mechanisms • Supplementary information

Table S2. P-values for Figure S1A

Figure S1A	Time Period	Stage of analysis	Genotype	Comparison	P value
	80-86 h	II Instar	*CS*	*park^{13}/+*	1
			CS	*itpr^{sv35}/+*	NC
			park^{13}/+	*itpr^{sv35}/+*	0.9999
			CS	*itpr^{sv35}/park^{13}*	0.00257
			park^{13}/+	*itpr^{sv355}/park^{13}*	0.01064
			itpr^{sv35}/+	*itpr^{sv35}/park^{13}*	0.00257
			CS	*itpr^{ka901}/+*	0.42165
			CS	*itpr^{ka901}/park^{13}*	0.2378
			itpr^{ka901}/+	*itpr^{ka901}/park^{13}*	0.15502
			park^{13}/+	*itpr^{ka901}/park^{13}*	0.3153
			park^{13}/+	*itpr^{ka901}/+*	0.57902
			itpr^{sv35}/+	*itpr^{ka901}/+*	0.42165
			itpr^{sv35}/park^{13}	*itpr^{ka901}/park^{13}*	0.06677
		III Instar	*CS*	*park^{13}/+*	0.42165

Disease Models & Mechanisms • Supplementary information

			CS	$itpr^{sv35}$/+	0.10119
			$park^{13}$/+	$itpr^{sv35}$/+	0.67787
			CS	$itpr^{ka9011}$/+	0.01613
			$park^{13}$/+	$itpr^{ka901}$/+	0.3739
			$itpr^{sv35}$/+	$itpr^{ka901}$/+	0.3739
	128-136 h	II instar	$itpr^{sv35}/park^{13}$	$itpr^{ka901}/park^{13}$	0.60865
		III instar	*CS*	$park^{13}$/+	0.27458
			$park^{13}$/+	$itpr^{sv35}$/+	0.00718
			CS	$itpr^{sv35}$/+	0.00106
			CS	$itpr^{ka901}$/+	0.00607
			$park^{13}$/+	$itpr^{ka901}$/+	0.00897
			$itpr^{sv35}$/+	$itpr^{ka901}$/+	0.34864
		Pupae	*CS*	$park^{13}$/+	0.64333
			$park^{13}$/+	$itpr^{sv35}$/+	0.01795
			CS	$itpr^{sv35}$/+	0.00797
			CS	$itpr^{ka9011}$/+	0.01347
			$park^{13}$/+	$itpr^{ka9011}$/+	0.02138
			$itpr^{sv35}$/+	$itpr^{ka901}$/+	0.76764
	176-182 h	III Instar	*CS*	$itpr^{ka901}$/+	0.189
		Pupae	*CS*	$park^{13}$/+	0.01006
			$park^{13}$/+	$itpr^{sv35}$/+	0.03347
			CS	$itpr^{sv35}$/+	0.34527
			CS	$itpr^{ka901}$/+	0.01006
			$park^{13}$/+	$itpr^{ka901}$/+	NC
			$itpr^{sv35}$/+	$itpr^{ka901}$/+	0.03347

Disease Models & Mechanisms • Supplementary information

Table S3. P- values for Fig S1C-F

**Fig S1C**	**Test - Mann Whitney**		
**Genotype**	**Comparison**	**Day**	**p-value**
$park^{13}/itpr^{ug3}$	*Canton S*	25	<0.0001
$nsyb > Itpr^{+}$ $(park^{13}/itpr^{ug3})$	*Canton S*	25	1.35E-04
$dmef > Itpr^{+}$ $(park^{13}/itpr^{ug3})$	*Canton S*	25	4.07E-04
$nsyb > Parkin^{+}$ $(park^{13}/itpr^{ug3})$	*Canton S*	25	<0.0001
$dmef > Parkin^{+}$ $(park^{13}/itpr^{ug3})$	*Canton S*	25	0.00646
$nsyb > Itpr^{+}$ $(park^{13}/itpr^{ug3})$	$park^{13}/itpr^{ug3}$	25	8.17E-04
$dmef > Itpr^{+}$ $(park^{13}/itpr^{ug3})$	$park^{13}/itpr^{ug3}$	25	0.2049
$nsyb > Parkin^{+}$ $(park^{13}/itpr^{ug3})$	$park^{13}/itpr^{ug3}$	25	0.07129
$dmef > Parkin^{+}$ $(park^{13}/itpr^{ug3})$	$park^{13}/itpr^{ug3}$	25	0.02678
**Fig S1D**	**Test - Mann Whitney**		
**Genotype**	**Comparison**	**Day**	**p-value**
TH/+	$TH > Parkin^{IR\text{-}KK104363}$	25	<0.0001
$Parkin^{IR\text{-}KK104363}/+$	$TH > Parkin^{IR\text{-}KK104363}$	25	1.26E-04
TH/+	$TH > Parkin^{IR\text{-}31259}$	25	0.03369
$Parkin^{IR\text{-}31259}/+$	$TH > Parkin^{IR\text{-}31259}$	25	0.15028
TH/+	$TH > Parkin^{IR\text{-}37509}$	25	<0.0001
$Parkin^{IR\text{-}37509}/+$	$TH > Parkin^{IR\text{-}37509}$	25	2.20E-04
**Fig S1E**	**Test - Mann Whitney**		
**Genotype**	**Comparison**	**Day**	**P-value**
$park^{13}/+$	$park^{13}/park^{1}$	25	<0.0001
$park^{1}/+$	$park^{13}/park^{1}$	25	<0.0001
**Fig S1F**	**Test - Mann Whitney**		
**Genotype**	**Comparison**	**Day**	**P-value**

Disease Models & Mechanisms • Supplementary information

pink1^{B9}/+	*Canton S*	5	0.75384
itpr^{ug3}/+	*Canton S*	5	0.61559
pink1^{B9}/itpr^{ug3}	*Canton S*	5	<0.0001
pink1^{B9}/+	*itpr^{ug3}/+*	5	0.97677

Table S4. P-values for Fig 2B

**Fly Brain**	**No. of DA neurons (PPL1 cluster)**	
	THD' > MitoGCaMP	***THD' > MitoGCaMP; park^{13}/itpr^{ug3}***
Brain_1	12	10
Brain_2	12	10
Brain_3	12	11
Brain_4	11	11
Brain_5	12	
**t-Test: Two-Sample Assuming Unequal Variances (two tailed)**		
P value	0.010067883	

Table S5. P-values for Fig S2C.

**Fig S2C**	**Test - Mann Whitney**		
**Genotype**	**Comparison**	**Day**	**P-value**
THD'/+; UAS SPLICS/+	*THD'/+; UAS SPLICS/ park^{13}*	25	0.43113
THD'/+; UAS SPLICS/+	*THD'/+; itpr^{ug3}/ UAS SPLICS*	25	0.01182
THD'/+; UAS SPLICS/+	*THD'/+ ; itpr ^{ug3}/UAS SPLICS, park^{13}*	25	0.00584
THD'/+; UAS SPLICS/ park^{13}	*THD'/+; itpr^{ug3}/ UAS SPLICS*	25	0.10476
THD'/+; UAS SPLICS/ park^{13}	*THD'/+ ; itpr ^{ug3}/UAS SPLICS, park^{13}*	25	0.04266
THD'/+; itpr^{ug3}/ UAS SPLICS	*THD'/+ ; itpr ^{ug3}/UAS SPLICS, park^{13}*	25	0.65221

Disease Models & Mechanisms • Supplementary information

Table S6. P-values for Fig 3 and S3.

**Figure 3A**	**Test: Mann Whitney**	
**Genotype**	**Comparison**	**P-value**
THD' > UAS GCaMP; park^{13}/itpr^{ug3}	*THD' > UAS GCaMP*	0.2586
**Figure 3B**	**Test: Mann-Whitney**	
**Genotype**	**Comparison**	**P-value**
THD' > MitoGCaMP	*THD' > MitoGCaMP;park^{13}/+*	0.90957
THD' > MitoGCaMP	*THD' > MitoGCaMP; itpr^{ug3}/+*	0.61613
THD' > MitoGCaMP	*THD' > MitoGCaMP; park^{13}/itpr^{ug3}*	0.55767
THD' > MitoGCaMP;park^{13}/+	*THD' > MitoGCaMP; park^{13}/itpr^{ug3}*	0.68289
THD' > MitoGCaMP; itpr^{ug3}/+	*THD' > MitoGCaMP; park^{13}/itpr^{ug3}*	1
THD' > MitoGCaMP;park^{13}/+	*THD' > MitoGCaMP; itpr^{ug3}/+*	0.33804
**Figure S3D**	**Test: Mann Whitney**	
**Genotype**	**Comparison**	**P-value**
THD' > MitoGCaMP	*THD' > MitoGCaMP;park^{13}/+*	0.95889
THD' > MitoGCaMP	*THD' > MitoGCaMP; itpr^{ug3}/+*	0.27258
THD' > MitoGCaMP	*THD' > MitoGCaMP; park^{13}/itpr^{ug3}*	0.99247
THD' > MitoGCaMP;park^{13}/+	*THD' > MitoGCaMP; park^{13}/itpr^{ug3}*	0.78437
THD' > MitoGCaMP; itpr^{ug3}/+	*THD' > MitoGCaMP; park^{13}/itpr^{ug3}*	0.22294
THD' > MitoGCaMP;park^{13}/+	*THD' > MitoGCaMP; itpr^{ug3}/+*	0.39828
**Figure S3F**	**Test: T-test one tailed**	
**Genotype**	**Comparison**	**P-value**
THD' > UAS GCaMP; park^{13}/itpr^{ug3}	*THD' > UAS GCaMP*	0.874419

Table S7. P-values for Fig 4.

**Fig 4**	**Test: Mann-Whitney**		
**Genotype**	**Comparison**	**Day**	**P-value**
THD' > UAS MitoQC DMSO	*THD' > UAS MitoQC; park^{13}/itpr^{ug3}* DMSO	25	0.03856

Disease Models & Mechanisms • Supplementary information

THD' > UAS MitoQC 20mM Paraquat	*THD' > UAS MitoQC* DMSO	25	<0.0001
THD' > UAS MitoQC; park^{13}*/itpr*^{ug3} DMSO	*THD' > UAS MitoQC* 20mM Paraquat	25	<0.0001
THD' > UAS MitoQC; park^{13}*/itpr*^{ug3} DMSO	*THD' > UAS MitoQC; park*^{13}*/itpr*^{ug3} 20mM Paraquat	25	<0.0001
THD' > UAS MitoQC 20mM Paraquat	*THD' > UAS MitoQC; park*^{13}*/itpr*^{ug3} 20mM Paraquat	25	0.51856
THD' > UAS MitoQC DMSO	*THD' > UAS MitoQC; park*^{13}*/itpr*^{ug3} 20mM Paraquat	25	<0.0001

Table S8. P-values of Fig 5 and Fig S5

**Fig 5A and Fig S5A**	**Test - Mann Whitney**		
**Genotype**	**Comparison**	**Day**	**P-value**
THD'/+;park^{13}*/+*	*Canton S*	25	0.10216
UAS Catalase/+;itpr^{ug3}*/+*	*Canton S*	25	0.91705
THD'/+;park^{13}*/+*	*UAS Catalase/+;itpr*^{ug3}*/+*	25	0.21074
park^{13}*/itpr*^{ug3}	*Canton S*	25	<0.0001
THD'/UASCatalase; park^{13}*/itpr*^{ug3}	*park*^{13}*/itpr*^{ug3}	25	<0.0001
THD' /UASCatalase; park^{13}*/itpr*^{ug3}	*THD' Gal4/+;park*^{13}*/+*	25	0.12237
THD'/UAS Catalase; park^{13}*/itpr*^{ug3}	*UAS Catalase/+;itpr*^{ug3}*/+*	25	0.84662
THD'/UAS Catalase; park^{13}*/itpr*^{ug3}	*THD' Gal4;park*^{13}*/itpr*^{ug3}	25	3.94E-04
THD'/UAS Catalase; park^{13}*/itpr*^{ug3}	*UAS Catalase; park*^{13}*/itpr*^{ug3}	25	<0.0001
THD'/UAS SOD2; park^{13}*/itpr*^{ug3}	*park*^{13}*/itpr*^{ug3}	25	0.61514
THD'/UAS SOD2; park^{13}*/itpr*^{ug3}	*THD'/+;itpr*^{ug3}*/+*	25	<0.0001
THD'/UAS SOD2; park^{13}*/itpr*^{ug3}	*UAS SOD2/+;park*^{13}*/+*	25	<0.0001

Disease Models & Mechanisms • Supplementary information

THD'/UAS SOD2; park^{13}/itpr^{ug3}	*UAS SOD2/+;park^{13}/itpr^{ug3}*	25	0.2597
THD'/+;itpr^{ug3}/+	*UAS SOD2/+;park^{13}/+*	25	0.84585
THD'/UAS Catalase; park^{13}/itpr^{ug3}	*THD'/UAS SOD2; park^{13}/itpr^{ug3}*	25	<0.0001
THD'/+;itpr^{ug3}/+	*Canton S*	25	0.78281
UAS SOD2/+;park^{13}/+	*Canton S*	25	0.489
THD' Gal4;park^{13}/itpr^{ug3}	*park^{13}/itpr^{ug3}*	25	<0.0001
UAS Catalase;park^{13}/itpr^{ug3}	*park^{13}/itpr^{ug3}*	25	0.01354
UAS SOD2/+;park^{13}/itpr^{ug3}	*park^{13}/itpr^{ug3}*	25	0.46391
UAS Catalase;park^{13}/itpr^{ug3}	*THD' Gal4;park^{13}/itpr^{ug3}*	25	0.08085
UAS SOD2/+;park^{13}/itpr^{ug3}	*THD' Gal4;park^{13}/itpr^{ug3}*	25	<0.0001
THD'/UAS SOD2; park^{13}/itpr^{ug3}	*THD' Gal4/+;itpr^{ug3}/+*	25	<0.0001
THD'/UAS SOD2; park^{13}/itpr^{ug3}	*UAS SOD2/+;park^{13}/+*	25	<0.0001
THD';park^{13}/itpr^{ug3}	*THD' Gal4/+;itpr^{ug3}/+*	25	2.49E-04
THD';park^{13}/itpr^{ug3}	*UAS SOD2/+;park^{13}/+*	25	<0.0001
UAS Catalase;park^{13}/itpr^{ug3}	*UAS SOD2/+;park^{13}/itpr^{ug3}*	25	0.00301
**Fig S5B**			
park^{25}/+	*TH/+*	10	0.82893
UAS itpr^{DN}/+	*TH/+*	10	0.67695
UAS itpr ^{DN}/TH	*TH/+*	10	0.18847
UAS itpr^{DN}/+; TH/+	*TH/+*	10	0.60958
Catalase/+; *park^{25}/+*	*TH/+*	10	0.76064
UAS itpr^{DN}/+; TH /park^{25}	*TH/+*	10	<0.0001
UAS itpr^{DN}; TH/Catalase; park^{25}	*TH/+*	10	0.00731
UAS itpr^{DN}; TH/park^{25}	*UAS itpr^{DN}; TH/Catalase; park^{25}*	10	0.01872
**Fig S5C**			
THD'/+; park^{25}/+	*THD'/+; park^{25}/itpr^{ug3}*	25	<0.0001
THD'/+; park^{25}/itpr^{ug3}	*THD'; park^{25}/Catalase^{+}; itpr^{ug3}*	25	<0.0001
THD'/+; park^{25}/+	*THD; park^{25}/Catalase^{+}; itpr^{ug3}*	25	0.02614
**Fig 5C**	**Test - Mann Whitney**		
**Genotype**	**Comparison**	**Day**	**P-value**
THD'/UAS mito roGFP Orp1;park^{13}/itpr^{ug3}	*THD'> UAS mito roGFP Orp1*	25	<0.0001

Disease Models & Mechanisms • Supplementary information

THD'/UAS mito roGFP Orp1; Itpr^{+}, park^{13}/itpr^{ug3}	THD'/UAS mito roGFP Orp1;park^{13}/itpr^{ug3}	25	<0.0001
THD'/UAS mito roGFP Orp1; Parkin^{+}, park^{13}/itpr^{ug3}	THD' /UAS mito roGFP Orp1;park^{13}/itpr^{ug3}	25	0.00114
THD'/UAS mito roGFP Orp1; Catalase^{+}, park^{13}/itpr^{ug3}	THD' /UAS mito roGFP Orp1;park^{13}/itpr^{ug3}	25	<0.0001
THD'/UAS mito roGFP Orp1; park^{13}/+	THD'/UAS mito roGFP Orp1;park^{13}/itpr^{ug3}	25	<0.0001
THD' /UAS mito roGFP Orp1; itpr^{ug3}/+	THD'/UAS mito roGFP Orp1;park^{13}/itpr^{ug3}	25	<0.0001
THD'/UAS mito roGFP Orp1; park^{13}/+	THD' > UAS mito roGFP Orp1	25	0.05625
THD' /UAS mito roGFP Orp1; itpr^{ug3}/+	THD' > UAS mito roGFP Orp1	25	0.00759
THD' /UAS mito roGFP Orp1; Itpr^{+}, park^{13}/itpr^{ug3}	THD' /UAS mito roGFP Orp1; Parkin^{+}, park^{13}/itpr^{ug3}	25	0.32728
THD' /UAS mito roGFP Orp1; Itpr^{+}, park^{13}/itpr^{ug3}	THD' /UAS mito roGFP Orp1; Catalase^{+}, park^{13}/itpr^{ug3}	25	0.36847
THD' /UAS mito roGFP Orp1; Parkin^{+}, park^{13}/itpr^{ug3}	THD' /UAS mito roGFP Orp1; Catalase^{+}, park^{13}/itpr^{ug3}	25	0.02759
THD' /UAS mito roGFP Orp1; park^{13}/+	THD' /UAS mito roGFP Orp1; itpr^{ug3}/+	25	0.87361
**Fig S5D and S5E**			
THD'/UAS mito roGFP Orp1;park^{13}/itpr^{ug3}	THD' > UAS mito roGFP Orp1	5	0.17746
THD' /UAS mito roGFP Orp1;park^{13}/itpr^{ug3+} - 5D	THD' > UAS mito roGFP Orp1-25D	5 vs 25	8.36433E-4
THD' /UAS mito roGFP Orp1;park^{13}/itpr^{ug3+} - 5D	THD' /UAS mito roGFP Orp1;park^{13}/itpr^{ug3+} - 25D	5 vs 25	<0.0001
THD' > UAS mito roGFP Orp1 - 5D	THD' > UAS mito roGFP Orp1-25D	5 vs 25	0.00108
THD' /UAS mito roGFP Orp1;park^{13}/itpr^{ug3}	THD' > UAS mito roGFP Orp1	25	<0.0001
THD' > UAS mito roGFP Orp1 - 5D	THD'/UAS mito roGFP Orp1;park^{13}/itpr^{ug3+} - 25D	5 vs 25	1.25219E-4

Table S9. Quantification data for Fig 6 and Fig S6

Figure 6

Sl. No.	Genotype	Compound	Conc. (ng/mL)	Ratio of Area (Compound/ISTD X Dilution factor)	Peak Area	Phenylalanine-ISTD (15.625 ng/mL) – Peak Area
1	*Canton S*	DOPAC	144.8052	0.27513	622.38	63339.73
2	*Canton S*		222.3685	0.4225	873.89	57914.31
3	*Canton S*		325.5193	0.618487	1339.2	60627.56
1	*park*^{13}/*itpr*^{ug3}		421.5035	0.800857	1271.8	44465.55
2	*park*^{13}/*itpr*^{ug3}		314.406	0.597371	1404	65807.09
3	*park*^{13}/*itpr*^{ug3}		498.3851	0.946932	2349.6	69474.85
1	*Canton S*	DA	31.02179	7.20841	11447.35	44465.55
2	*Canton S*		18.04752	1.424653	3348.295	65807.09
3	*Canton S*		37.665	1.668201	4139.214	69474.85
1	*park*^{13}/*itpr*^{ug3}		64.3608	3.47444	7859.647	63339.73
2	*park*^{13}/*itpr*^{ug3}		12.72012	2.021322	4180.839	57914.31
3	*park*^{13}/*itpr*^{ug3}		14.89465	4.21848	9134.148	60627.56

Figure S6

Sl. No.	Compound	Conc (ng/mL)	Ratio of Area (Compound/ISTD)	Peak Area	Phenylalanine-ISTD (15.625 ng/mL) – Peak Area
1	DOPAC Calibration	0.977	0.000081	3.0423	37256.34
2		1.953	0.0028	105.31	37628.63
3		3.906	0.0068	246.19	36247.73

Disease Models & Mechanisms • Supplementary information

4		7.813	0.0143	548.51	38414.48
5		15.625	0.0322	1162.6	36135.30
6		31.250	0.0668	2344.8	35090.95
7		62.50	0.1286	4481.4	34847.32
8		125	0.2250	7766.8	34515.92
9		250	0.4680	16466	35185.03
1	DA Calibration	0.1220	0.0126	512.921	40648.74
2		0.2441	0.0248	996.739	40168.14
3		0.4883	0.0496	1929.346	38916.26
4		0.9766	0.1126	4195.453	37256.34
5		1.9531	0.2232	8428.704	37628.63
6		3.9063	0.4543	16468.18	36247.73
7		7.8125	0.8781	33731.03	38414.48
8		15.625	1.8692	67542.97	36135.3
9		31.25	3.8889	136466.3	35090.95
10		62.5	7.8382	273139.8	34847.32
11		125	14.776	509995.6	34515.92
12		250	27.359	962614.3	35185.03

Table S10. Statistics for Fig. 6

Genotype	Compound	Sample mean ng/ml (N=3)	Std. Deviation	P value (t test)
Canton S	DOPAC	230.9	±74	0.04
park^{13}/*itpr*^{ug3}	DOPAC	411.4	±75	
Canton S	DA	28.9	+ 8	0.9
park^{13}/*itpr*^{ug3}	DA	30.6	+ 23	

Table S11. P- values for Fig 7 and Fig S7

**Figure 7D**	**Test - Mann Whitney**	
**Genotype**	**Comparison**	**P-value**
THD' > Perceval; park^{13}/itpr^{ug3}	*THD' > Perceval*	8.30E^{-04}
THD' > Perceval; Itpr^{+}, park^{13}/itpr^{ug3}	*THD' > Perceval*	0.04547
THD' > Perceval; Parkin^{+}, park^{13}/itpr^{ug3}	*THD' > Perceval*	0.00135
THD' > Perceval; Catalase^{+}, park^{13}/itpr^{ug3}	*THD' > Perceval*	0.08166
THD' > Perceval; Itpr^{+}, park^{13}/itpr^{ug3}	*THD' > Perceval; park^{13}/itpr^{ug3}*	0.04296
THD' > Perceval; Parkin^{+}, park^{13}/itpr^{ug3}	*THD' > Perceval; park^{13}/itpr^{ug3}*	0.2319
THD' > Perceval; Catalase^{+}, park^{13}/itpr^{ug3}	*THD' > Perceval; park^{13}/itpr^{ug3}*	0.12106
THD' > Perceval; Itpr^{+}, park^{13}/itpr^{ug3}	*THD' > Perceval; Parkin^{+}, park^{13}/itpr^{ug3}*	0.13159
THD' > Perceval; Itpr^{+}, park^{13}/itpr^{ug3}	*THD' > Perceval; Catalase^{+}, park^{13}/itpr^{ug3}*	0.93944
THD' > Perceval; Parkin^{+}, park^{13}/itpr^{ug3}	*THD' > Perceval; Catalase^{+}, park^{13}/itpr^{ug3}*	0.49959
**Figure S7B**	**Test - Mann Whitney**	
**Genotype**	**Comparison**	**p-value**
THD' > Perceval;park^{13}/+	*THD' > Perceval*	0.34078
THD' > Perceval;itpr^{ug3}/+	*THD' > Perceval*	0.19599
THD' > Perceval;park^{13}/+	*THD' > Perceval;itpr^{ug3}/+*	0.29414

Disease Models & Mechanisms • Supplementary information
